# Quantifying the global film festival circuit: Networks, diversity, and public value creation

**DOI:** 10.1371/journal.pone.0297404

**Published:** 2024-03-06

**Authors:** Vejune Zemaityte, Andres Karjus, Ulrike Rohn, Maximilian Schich, Indrek Ibrus

**Affiliations:** 1 Baltic Film, Media and Arts School, Tallinn University, Tallinn, Estonia; 2 ERA Chair for Cultural Data Analytics, Tallinn University, Tallinn, Estonia; 3 School of Humanities, Tallinn University, Tallinn, Estonia; 4 Estonian Business School, Tallinn, Estonia; The Chinese University of Hong Kong, HONG KONG

## Abstract

Film festivals are a key component in the global film industry in terms of trendsetting, publicity, trade, and collaboration. We present an unprecedented analysis of the international film festival circuit, which has so far remained relatively understudied quantitatively, partly due to the limited availability of suitable data sets. We use large-scale data from the Cinando platform of the Cannes Film Market, widely used by industry professionals. We explicitly model festival events as a global network connected by shared films and quantify festivals as aggregates of the metadata of their showcased films. Importantly, we argue against using simple count distributions for discrete labels such as language or production country, as such categories are typically not equidistant. Rather, we propose embedding them in continuous latent vector spaces. We demonstrate how these “festival embeddings” provide insight into changes in programmed content over time, predict festival connections, and can be used to measure diversity in film festival programming across various cultural, social, and geographical variables—which all constitute an aspect of public value creation by film festivals. Our results provide a novel mapping of the film festival circuit between 2009–2021 (616 festivals, 31,989 unique films), highlighting festival types that occupy specific niches, diverse series, and those that evolve over time. We also discuss how these quantitative findings fit into media studies and research on public value creation by cultural industries. With festivals occupying a central position in the film industry, investigations into the data they generate hold opportunities for researchers to better understand industry dynamics and cultural impact, and for organizers, policymakers, and industry actors to make more informed, data-driven decisions. We hope our proposed methodological approach to festival data paves way for more comprehensive film festival studies and large-scale quantitative cultural event analytics in general.

## Introduction

Over 12,000 film festivals happen around the world each year [1], ranging from major industry events like Cannes or Sundance to medium, small, and even tiny local events [2]. Festivals differ in sizes of their budget, programs, and audiences, as well as profiles, spheres of action, and specializations [2, 3]. They form a global festival circuit, a vital, organic part of the film industry, interconnected via the flow of films (participating in multiple consecutive festivals) and characterized by emerging complex network structures of individuals, companies, and events [4].

This paper presents an unparalleled quantitative analysis of the global film festival network. We consider a film festival a planned event [5] that follows procedures for curation, regulation, and selection to program films and audiovisual works (including television content and shorts) to be screened to broad audiences ranging from the general public to industry professionals and press, during a specified period in a prior defined place [6]. Following the industry response to the COVID-19 pandemic, we expand the definition to include online editions of previously established festival series.

Film festivals are seen as “key assets” and “an essential link in the film industry ecosystem” [7] because of their unique ability to determine, reproduce, and contest multiple types of values for a variety of agents [8]. In exchange for national funding and granted subsidized local infrastructures like archives or educational institutions, festivals generate both economic and symbolic capital for their host countries, as well as engage in social development activities and purposes, such as training, educational programs, and commitment to cultural diversity [9, 10]. The economic value is primarily derived from the expenses of organizing the festival and the spending brought in by visitors to the host regions, which totaled over 118M US$ for Utah during Sundance 2023 [11], while over 24M CHF for the Ticino region during Locarno 2022 [12]. Every Swiss franc invested in the Locarno film festival has been estimated to generate a return of 3 francs for the region [13]. Festivals also create employment opportunities, with 1,608 jobs created by Sundance 2023 [11] and 1,040 jobs by Locarno 2019 [13]. However, the fairness of pay for festival staff has been recently questioned [14]. Additionally, media coverage during festivals creates symbolic value for the host region, estimated at over 5M CHF, with over 15,000 domestic and international media features for Locarno 2019 [13].

For filmmakers and films, festivals generate symbolic cultural and marketing value by creating global media exposure around granted prizes, participation in competition programs, and inclusion in programming [8, 10, 15, 16]. For instance, festivals accredited by the International Federation of Film Producers Associations (FIAPF) cumulatively held the world premieres of nearly 1,700 films, granted 32,000 press badges, and generated 4.5M admissions in 2017 [17]. Festivals also generate economic value for the film industry by facilitating investment in productions via development programs and creating an array of business opportunities via markets, such as for signing co-production deals, selling distribution rights, or arranging licensing [7, 8, 10, 15]. For example, the largest industry event, the Cannes Film Market 2023, held 1,500 market screenings and attracted around 14,000 accredited professionals and more than 300 sales companies, hosting 510 exhibitors and 60 national pavilions [18]. Festivals, however, have different weights in their capacity to generate value [19]. In that regard, festivals have been ranked in hierarchies [4, 15], grouped into the center and periphery [20, 21], or business versus audience events [22]. Still, the festival circuit is where key film industry trends are negotiated, initiated, and mirrored. Understanding film festivals, therefore, contributes to a wider and more detailed understanding of the global film industry.

While the global film industry has attracted numerous quantitative, data-driven studies (see [23] for an overview from movie economics), international film festivals surprisingly remain quantitatively understudied [8, 24], except for recent contributions [25–27]. Most film and media research on festivals employs qualitative methods, including textual, ethnographic, and archival analyses [24], or produces monographic case studies, with a clear need for conceptual generalizations and systematic comparative analysis of film festivals [8]. This lack of quantitative, comparative approaches in film festival studies is partially due to the limited availability of systematically collected data. Festival databases employed in previous research have been constrained based on festival specialization, i.e. queer festivals [28], or festival location, i.e. American festivals [29] or Chilean festivals [30], or were collected using selective techniques [26]. While multiple platforms have been created to facilitate the operations of the film festival industry in recent years (i.e. Cinando [31], b.square [32], Eventival [33], Eventive [34]), academic research so far has not utilized their data.

A need for more sophisticated methods has also been identified within the broader field of festival and event research [35]. Although, according to a large-scale literature review [36], quantitative methods were more prominent than qualitative in this broader field, the quantitative approaches have been primarily based on national cross-sectional data collected via audience surveys rather than employing granular event data directly, with an identified need for analyses from a broader range of regions and adopting longitudinal approaches. In particular, there has been an interest in understanding “how events can engage emerging markets and deal with cross-cultural differences” [37]. However, limiting research to positivist quantitative methods has also been cautioned against [38], calling for theory development in addition to the empirical [35–37].

The contribution of our work concerning the identified research gaps is threefold: we propose using a so far unexplored data source for studying film festivals, develop and apply suitable quantitative methods, and suggest relevant theories to ground and interpret the findings. This paper offers a first-of-its-kind quantitative, longitudinal, and cross-regional study of film festivals based on 13 years of systematically collected programming data. The work is enabled by unprecedented access to the Cinando database of the Cannes Film Market (also known as Marché du Film—Festival de Cannes). We apply network analysis to the festival circuit and adapt a vector embedding approach to operationalize festivals as quantitatively comparable units. This allows for data-driven metric characterization and visualization of the global festival landscapes, quantification of longitudinal trends, and measurement of diversity in film festival programming across various social, cultural, and geographic variables. The findings are interpreted using theories of public value generation and cultural diversity.

The remainder of this section presents the theoretical framework of our endeavor. The Methods section introduces our data and methodological approach, followed by Results, in light of the industry context and previous research, a final Discussion reflecting on our findings, and Conclusions.

### Public value theory for studying film festivals

As events and institutions, film festivals are typically, to a significant extent, publicly funded and have a public purpose. Hence, we approach the subject using the theories of public value and discuss festivals as institutional creators of value in the wider film industry. Within the public administration studies domain, “public value” refers to the ways in which public agencies and institutions participate in the broader value-creation processes, especially in the public interest [39]. For media and cultural institutions, this could play out in different ways. First, by facilitating a functioning public sphere where what is valued by different publics can be sorted out. Second, by delivering to the publics the information and experiences they value the most. Third, by re-conceptualizing the relevant markets as “innovation systems” [40]. This enables addressing their coordination failures and facilitating their evolution so that these broader markets start providing the values appreciated by the publics, leading to the feasible evolution of societies and general well-being. Altogether, the contemporary public value thinking perceives that there are multiple publics dispersed across the media system [41]. What is valued by these publics is always contextual and emergent. This is why a systemic and holistic approach is necessary to address the interconnections between different media and cultural institutions, facilitating the emergence of those values, media texts, and forms in the right contexts, thus serving the public interest and empowering people in leading their lives [42].

A new way for exploring the creation of public value by the media and cultural industries has emerged, which builds on innovation system studies [43–45]. Innovation system studies point to the emergence of innovations (also novel values) as a result of complex dialogic and knowledge exchange practices. Relatedly, any innovation system functions better if it is institutionally diverse because different institutions are driven by different epistemic communities and have different goals [46]. Working towards different interests increases the diversity within a system leading to a larger variety in its output. Hence, an institutionally diverse system is more resilient to risks and crises, being ready to move towards a wider range of alternative directions. Similarly, cultural diversity is important as it enriches the cultural meaning space of wider populations. Being exposed to an enriched cultural environment empowers people to become more creative and flexible in leading their lives in complex, dynamically changing, and globally interconnected cultures and societies [47].

Media and cultural institutions can serve a diverse cultural environment as public value to individuals, society, and industry. Often these dimensions are not only complementary but also mutually conditioning. For instance, film festivals facilitate cultural dialogues and contacts between public and private agencies internationally and curate the exposure of culturally diverse films to different local or national audiences. They constitute a marketing platform for film industries and function as an important interface for coordination and dialogue between different industry players and audiences. Yet, there has been a general concern that the focus on public value generation has been diminishing in the film industry in recent years [48]. We suggest that this critique has overseen the role of film festivals in constituting not only an enduring pillar of film policy to serve the public interest but also in embodying an evolving compromise between public and industry interests, which, in combination, produce “dynamic public value” or value for different beneficiaries [49]. This paper is motivated by the interest in better understanding the public value that film festivals create for different beneficiaries.

In this paper, we focus on the public value generation at the level of society, more specifically, the value of diversity created via film festival programming. We approach diversity as a multidimensional, multifaceted phenomenon. A festival’s identity and orientation are defined through programming [8]. Although the general understanding is that films seek out festivals, the festivals also need films to facilitate the construction of their identities [8, 50]. Programming is a complex task to find a compromise between satisfying artistic inspirations and activist intentions versus the audience tastes while balancing between too little variation and too much choice [51], and simultaneously considering outreach and practical limitations [2], such as the temporal event sequence, premiere requirements, etc. Having control over programming, however, also enables festivals to sidestep dominant and streamlined commercial distribution patterns and quickly respond to topical issues, in turn enriching the public sphere and democratizing audience access to films [52]. The latter function is especially important in terms of the localities where festivals operate since the main reason festivals are typically publicly funded is that they serve national communities [10]. Yet, the global media exposure generated by press representatives at the events also enables festivals to curate and diversify the international discourse on audiovisual culture by highlighting and legitimizing specific films and filmmakers, including from countries with limited production capacities, in their programming [16, 19, 52]. Since festivals have the potential to generate value for both the local and international public through their programming choices, we see programming diversity as a topical research question.

### Film festival diversity

Following the preceding discussion, we focus on diversity in film festival programming as a marker of public value. Diversity has been researched in a variety of film industry contexts, including program diversity in television [53], cultural diversity in film [54, 55], and gender diversity in film production [56], distribution [57], and festivals [26, 58].

Diversity can be measured across the dimensions of variety, balance, and disparity (as well as a combination of the three) [59]. Variety is relatively easy to assess since it concerns the number of categories in a system, for instance, the number of programmed film languages. When used alone, however, it fails to capture the full nuance of diversity, namely, how much the categories differ from each other, which is measured by disparity. Here, we primarily measure diversity via disparity, considering a festival to be diverse if it programs films of different themes, from distant countries or languages.

In our application, we differentiate between “internal” festival diversity and “external” festival circuit diversity [60, 61], as well as contributing diversity of each festival (how much it differs from the mainstream). Internal diversity is assessed at a micro level or within a subject, while external diversity is measured at a macro level or between subjects [61]. Following this logic, we define micro-level or internal diversity referring to the diversity within a single festival’s programming: a festival with high internal diversity features films highly different from one another (across some categories of interest). The festival circuit is high in macro-level diversity when films programmed across the circuit are highly variable. Finally, we also quantify how far a given festival is from the circuit latent average or the mainstream. A festival is interpreted to be contributing to the circuit diversity when its films are atypical or different in some aspect from the mainstream of the festival network, thereby enriching it.

Source diversity concerns the “off-screen” conditions in how a film is produced (by whom, where), such as its origin country, while content diversity addresses the “on-screen” film attributes, like genre distinctions [62]. The two are often not independent of each other [54]. We use film production countries and the gender of film directors and producers to evaluate aspects of source diversity and film content tags and languages to assess content diversity in festival programming.

## Methods and materials

**Fig 1:**
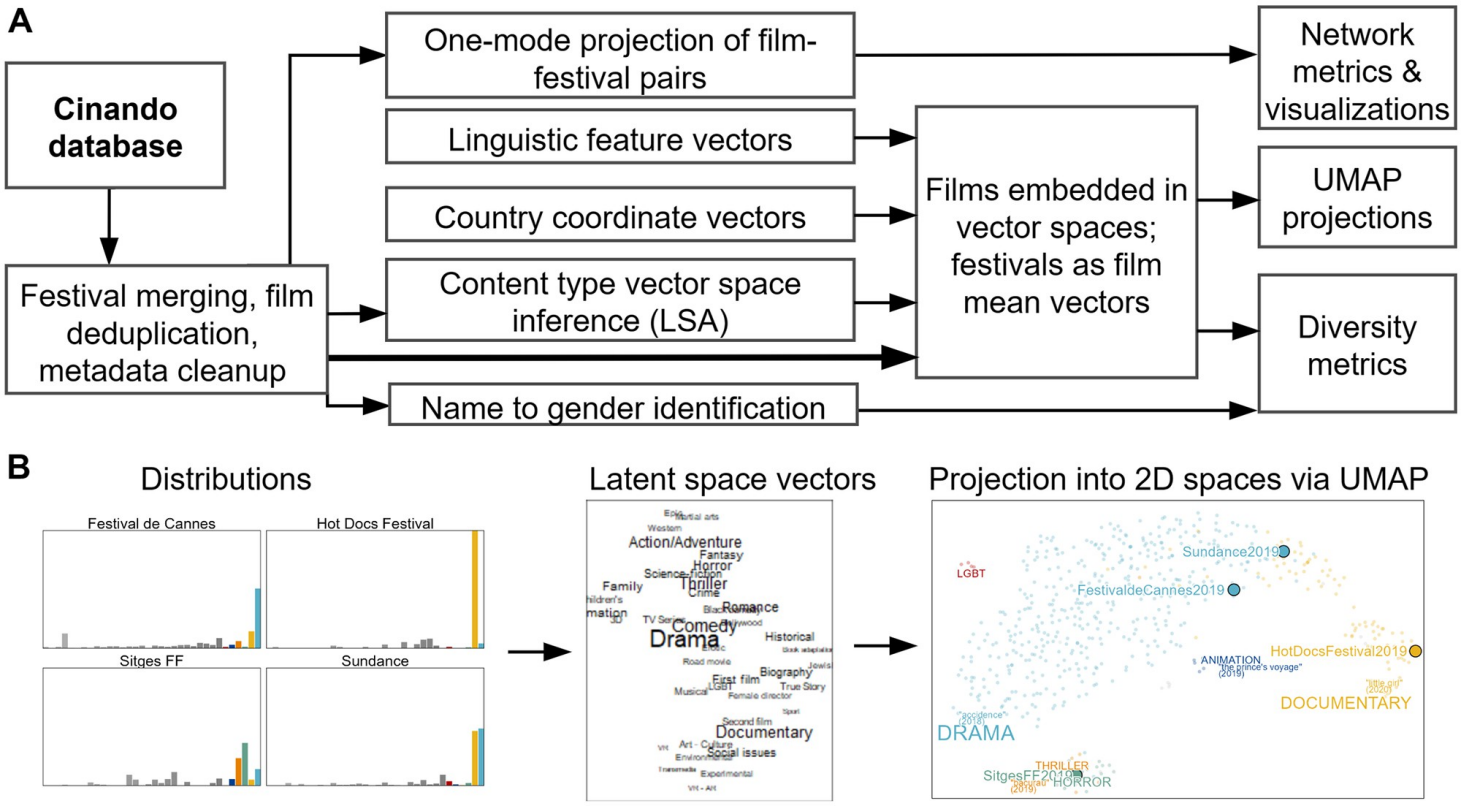
Project pipeline. (A) Data processing and analysis workflow as performed in this study. (B) Frequency distributions of data associated with individual festivals are transformed into latent space vectors, forming the basis for analytic results as reported in the text and for indicative projections into 2D spaces visualized using UMAP.

The first part of this section introduces the Cinando database employed as our primary data source, discussing the challenges and opportunities associated with such data access. We then propose two broad quantitative approaches based on film–festival co-occurrence for the analysis of this data, namely network analysis and vector embedding techniques, with a particular focus on measuring aspects and expressions of diversity. Our data processing and analysis workflow is summarized in Fig 1. The processed and enriched data is made available [63].

### Cinando: Data on international film festivals

This research has been made possible by data provided directly to us from the Cannes Film Market, the company operating the Cinando website and database. Launched in 2003 as the database of the attendees at the Cannes Film Festival, Cinando has since grown into the premier platform supporting hundreds of film festivals and film markets (industry events held during festivals, mostly oriented to promoting investment opportunities, rights sales, and production services). Cinando offers film professionals tools to navigate the film industry, including information about contacts, films, projects in development, market screening schedules, market attendees, and screeners. The platform services film festivals and markets by facilitating especially rights sales and investments, as well as business-to-business video on demand. The service is similar to that of the Festival Scope Pro platform (cf. [64]), except Cinando records festival programming data exhaustively rather than curating a list of festival films. The platform relies on a large proprietary relational database.

#### Data processing

Cinando’s data which concerns film festival programming, contains, at face value, 77,398 films programmed at 38,367 festival events, resulting in 183,865 film–festival event pairs between 2007–2022. The festival metadata includes event and, occasionally, festival series title, event location country, and event year (the absence of specific dates limits our analyses to yearly precision). Film metadata contains runtime, production year, origin countries, names of crew members, languages spoken in the film, and thematic or content type tags (labeled as film “kind” in the database). The latter is a mixture of tags typically used to describe films within the festival context, including genre (e.g. drama, documentary), target audience (e.g. children’s, family), identity (e.g. Jewish, LGBT), and production type (e.g. TV Series, VR). Some of these tags might be opinionated (e.g. LGBT rather than LGBTIQ+), but in this work, we describe and present this aspect of the database as is. Towards justifying the feasibility of our analysis, it makes sense to acknowledge that the inherent data set bias is both a limitation (see Limitations of our study section) and also a signal. In principle, the Cinando data set can be seen as a proxy for a collective perception of the film festival circuit, reflecting its diversity. While the ground truth of the total global festival circuit may be impossible to capture (at this point in time), our study nevertheless provides an unprecedented perspective into the film festival circuit diversity. While working with the best available data, our study already provides concrete and important opportunities to raise the associated public value at the level of individual festival organization and towards more comprehensive data collection and analysis, which will eventually overcome biases identified within and in reaction to our study.

The Cinando database contains a considerable number of duplicate entries, and missing values are highly prevalent. Films are often entered several times throughout their production and festival cycles, with differing amounts of metadata, differing runtimes, production credentials, years, and even different titles. Festivals are also often entered under several alternate spellings. For example, the Cannes Film Festival itself exists in the database also as Festival de Cannes, Cannes Festival, Cannes IFF, Cannes International Film Festival, and many other variations.

Our goal to compare and analyze festivals necessitated first cleaning and homogenizing the data to make it usable. While some spelling variations in event titles such as spaces and case are easy to solve, the usage of various abbreviations and multilingual titles complicates matters. To merge such entries and their programmed films into coherent festival event units, we performed two machine-assisted cleaning iterations (see S1 Table for data disambiguation summary). This consisted of extracting a set of festival series titles, finding the top-100 event title strings most similar to each series (ranked by restricted Damerau-Levenshtein edit distance [65]), and manually verifying and merging these matches.

As Cinando’s data grew primarily via the creation of film and person entries associated with festivals, festival programs were often captured incompletely. After removing duplicate film entries (using title and production year), we only included festivals with at least 15 unique films in their programming. Although we base it on policy guidelines [3], this is an arbitrary threshold that balances between including smaller festivals and partial entries and leaving enough data to construct reasonably reliable festival profiles and distributions. When deduplicating films within festivals, we rank them by the number of tags in the target metadata variable (e.g. production countries) and the presence of production year, keeping the most informative entries. After cleaning, merging, and applying this threshold, 31,989 unique films spread across 616 festivals are left (including 28 one-off events and 588 events belonging to 102 identifiable festival series (i.e. 2 or more events)). This totals 41,483 film–festival data points. For the analyses based on a particular film metadata variable (crew gender, thematic type, production countries, film language), these numbers are slightly smaller, as not all films have all the metadata, meaning some festivals further fall under the 15 threshold and are excluded. The sheer size of the data set necessitates some automation, which can lead to errors and noise in the data, even with selective human verification. Given time and resource constraints, we accept the possible risk of both over-merging (incorrectly recording different entities as one) and undermerging (retaining duplicate entries). It is hoped that cleaner data sets will be available in future festival research.

In addition to cleaning the data, we carried out data enrichment in the form of marking festivals as being A- or B-list events, based on the FIAPF accreditation system [66]. While festivals have to apply for accreditation each year, we extrapolated the same ranking to all events in a series (i.e. Cannes 2014, Cannes 2015, see festival name glossary in S2 Table) based on the latest available FIAPF report [67]. This is a simplification due to detailed historical accreditation data not being easily available, and we make the assumption that festivals rarely switch between the lists. We acknowledge that the FIAPF ranking is not perfect and have indeed received industry criticism for its strict accreditation criteria, including ranking Sundance as a B-list event [68] despite it being considered one of the “big five” most prestigious film festivals globally, next to Venice, Cannes, Toronto, and Berlin [69]. However, we relied on FIAPF as the only available concrete list that reflects the hierarchical positions of festivals and is used by the film industry as a quality and reliability standard for producers, distributors, and sales agents.

We also enriched film data with the gender of the director and producer based on the first names of the crew listed on the Cinando database. We used a large (*N* = 694, 410) gendered names database [70] derived from Wikipedia to match names with binary gender. The latter is a limitation of the database; we acknowledge that gender exists on a multidimensional continuum. However, not all names are in the database, and some names are marked as unisex (e.g. *Robin*). We take this into account when calculating gender percentages. We provide bootstrapped 95% confidence intervals for the various diversity measures; in the case of gender diversity, the uncertainty of the name classifier is incorporated into the bootstrapping: the upper bound assumes all unknowns are women, and the lower assumes they are men.

Data cleaning and homogenizing were also required for both language and production country metadata due to many non-standard and misspelled entries. This was necessary to be able to match them with standardized country and language names, which was required for matching with external databases, as described below.

### Network connectedness and diversity

The international film festival circuit has been long discussed as a network in theory [4, 15, 52, 71], and described by borrowing terminology from network science, such as “a network with nodes” [52] or “dense network” [71]. Network methods have been applied to the broader domain of film, such as to study collaboration between creatives in film production [56, 72–74] or facilitate the discovery of films via multi-layer network models [75]. So far, however, the applications of network analysis to film festival data have been limited in either regional scope [76] or the time period of a single year [26].

Our network approach relies on film-in-festival co-occurrence. We project the bipartite co-occurrence data as a one-mode festival network, where festivals are linked if they share a film in their programming (cf. [77]). This enables using node degree centrality (the number of links a festival has) both as an indicator of connectedness but also as a complementary estimate of how much diversity an event contributes to the festival circuit. We use undirected networks, as data on when a festival takes place is limited to years in the Cindando data set. Directed networks linking events in temporal order could be constructed in future research if more precise event dates are available.

Since festival entries in the Cinando database are assumed to include partial or incomplete programs, and festivals vary naturally in the size of programming, we normalize the degree statistic by dividing it by the base-10 logarithm of the number of films in a given entry. This operation largely decorrelates festival entry size and degree while retaining a similar scale. Importantly, the network statistics are only valid and computed here for the middle time period (2015–2019) due to the shape of the whole data set (2009–2021). Given the roughly two-year life-cycle of festival films (see Festival networks section), events in the first and last years have fewer possible festivals to link to, and the data set itself is much smaller in the early years, translating to a lower but unknown baseline chance of being linked.

We use network methods to address the following research questions about the international film festival circuit: What are the structure and temporal dynamics of the international film festival network? How diverse is the programming of different festivals? Are there differences between the A- and B-list events? What factors predict connections between festivals?

### Quantifying festivals using metadata embeddings

While the network approach connects festivals based on sharing the same film in programming, the metadata embedding approach described in this section uses the characteristics of programmed films (content tags, production countries, and languages) to create comparable festival profiles. Such profiles could also include festival metadata, if available. We use film data because we are primarily interested in the diversity of festival programming. This is not to say these dimensions necessarily cover all possible aspects of the diversity of a festival or its programming, nor that they may not overlap.

Films could also be embedded in vector spaces using visual machine learning models or textual models applied to synopses or scripts. Indeed, natural language processing methods have been used to computationally study and classify films [78–81], film reviews, being an important industry feedback component [82–84], social media coverage of festivals [85], and gender bias in synopses and scripts [86]. Inferring and making use of film metadata and characteristics [87], as well as viewer activity [88, 89], has been a central interest of recommendation systems research, more so with the commercial importance stemming from increasing platformization, and digitalization of the film industry, in general [90]. In contrast to most of the aforementioned research and to the survey-focused event and festival research [9], here we focus on festivals as the primary unit of analysis but use available film metadata to construct festival profiles.

#### From count vectors to latent spaces

The easiest way to create comparable festival profiles or distributions would be to use (normalized) counts of metadata tags (of some variable of interest) of the films in a given festival program. In diversity literature, this has been operationalized as variety [59]. For example, to construct a thematic profile, the distribution of dramas, comedies, westerns, etc. in festivals could be used. For operationalizing concepts with only two categories like gender diversity, percentages or ratios of counts suffice.

However, we argue that using count distributions is not the best approach in most multinomial cases, as such metadata categories are typically not inherently equidistant. This, in turn, can bias further comparisons and derived measures, including those of complexity and diversity. Consider, for example, two years of a hypothetical festival: in the first year, it shows films from Norway, Sweden, and Denmark, and in the second year from Japan, Morocco, and Mexico, in the respective languages. If one were to simply count the number of different countries, one would reach the conclusion that the festival did not change in geographic or language diversity.

This would not be a satisfactory result for our purposes, however, as we are also interested in how diverse or variable the programming of a festival is. The solution to the lack of equidistance between categories (such as countries) is to embed all such metadata in suitable continuous spaces which reflect distances between categories. For the geographic or spatial example, the straightforward option for such space is to simply use the longitude and latitude of the countries (we use capital city coordinates, except Los Angeles for the US), and for distance metric, the shortest path geodesic (ellipsoid) distance. In this new continuous vector space, the hypothetical festival above, programming only Nordic films (from nearby coordinates), looks clearly less geographically diverse than the global one. This is analogous to using phylogenetic distances when dealing with categorical species [59] or co-occurrence-based measures in product space analysis [91]. We do not source distances but rather spaces where we can calculate various distances and diversity metrics.

For languages spoken in films, we use another externally-sourced space, the linguistic feature vectors from a typological database [92], compressed using Singular Value Decomposition to a lower dimensional space (the original vectors consist of hundreds of partly collinear features). In this space, closely related languages such as Czech and Slovak, or Urdu and Hindi, are close together, while unrelated and distant languages are far apart (S1 Fig).

For thematic content type, there is no external space that could be easily plugged in, but it can be inferred directly from data. Many films in the Cinando database have more than one thematic tag. We infer the thematic space using the thematic tag co-occurrence information, analogously to how word vectors are inferred in Natural Language Processing (NLP). The rationale goes back to the notion of “you shall know the word by the company it keeps” [93]. If two content tags (words) A and B occur on similar (but not necessarily the same) films sharing other tags (context), then A and B are likely similar in content description (meaning or function). The method transfer of distributional approaches from text applications to other data types has previously also proven useful in other fields, e.g. science of science [94] and social network analysis [95]. Since the number of unique content tags and the amount of film data are fairly small (in NLP terms), we use a simple model with few hyperparameters, Latent Semantic Analysis [96]. It consists of weighting the co-occurrence count matrix of the content tags (we use Positive Point-wise Mutual Information), followed by Singular Value Decomposition. Unlike word embeddings in NLP, there is no standard test set that we could use to measure the semantic quality of the resulting thematic space, but we are sufficiently satisfied with its structure based on manual inspection (S2 Fig). There, “thriller” is the closest tag for “horror”, “social issues” for “documentary”, “children’s” for “animation”, etc.

Unlike country and language, the thematic vector space is also operationalized slightly differently: if a film has multiple content tags (e.g. “romance, comedy”), then we average the respective vectors into a single composite latent vector. The rationale is that the content specification can be more than the sum of its parts. However, this would not make sense in the case of the language and geographical spaces (averaging the coordinates of a co-production between France and Japan would make it look like a film was made in Russia).

Using data-driven categories allows us to sidestep discussions on the “real” meaning of thematic content types, which include a mix of genre, target audience, creator’s identity, and production type tags, as well as the political question of language versus dialect. While we use the original tags in the graphs and text for easy reference, all underlying calculations are done on the latent spaces. We use Euclidean distance as the metric for the linguistic and thematic spaces.

The latent space approach not only allows for further quantification but also enables us to address the following research questions: How are international film festivals positioned in relation to one another across the thematic, geographic, and linguistic spaces? Do they form meaningful groups?

#### Latent spaces enable measuring similarity and diversity

We can now operationalize festivals as (weighted) mean vectors of their programmed films. A film in the Cinando database can have multiple ordered tags in a category such as production country. The order may carry different meanings in different film industry instances. Distributors might strategically choose which content tag to use first to better target specific audiences, countries could be ordered by the size of their contribution to production, with lead and co-producing countries listed before associated producers. Since we are interested in diversity, we consider all tags equally instead of attempting to come up with ordered weighting schemes suitable for different instances. We assign each tag an equal weight for the purposes of calculating festival vectors, as one over the number of tags on a film entry in a given category. For example, a film co-produced between the UK and the US would get 0.5 weights for each country tag, while a film made just in the UK would get a full weight of 1 for its single vector.

In each metadata space, tags or categories have their own vectors, films consist of multiple tag vectors (or one averaged vector in the case of thematic type)—and festivals are weighted means of their film vectors. Therefore, all three entities (tags, films, and festivals) for a given metadata category exist in the same continuous multidimensional latent space and are directly comparable. It is possible to calculate the similarity between any two festival vectors or run dimension reduction algorithms like Uniform Manifold Approximation and Projection (UMAP) [97] on the vector spaces to display them as two-dimensional graphs. This provides a birds-eye view of the festival circuit according to any of the metadata aspects (see Festival latent spaces section).

This approach also enables operationalizing diversity directly in the latent space without the need for operating around pairwise or nearest neighbor distances (cf. disparity in [59]). We use a mathematically simple metric analogous to Mean Absolute Deviation around the mean. For a given festival in a given metadata space, we first calculate its average latent vector (weighted as described above) and then average the distances of all its film vectors from that (again, weighted as above), using geodesic distance for country coordinates, and Euclidean distance in other spaces. This estimates the diversity within a festival: the more spread around the mean the film vectors, the more diverse a given festival is in a given space. We will refer to this as “internal diversity” (cf. [60, 61]). This can also be thought of more generally as a measure of complexity.

We also operationalize “contributing diversity”, a measure of distance from the mainstream, which may be interpreted as diversity a festival event contributes to the circuit as a whole. It is the average distance of all films in a given festival program from the grand mean vector of the festival circuit in our database. The latter is constructed as a weighted average of all films, where the weight is defined as the metadata weights of each film multiplied by one over the number of films in a festival. This assures all festivals count equally towards the mean, regardless of the number of films in their entries. Low contributing diversity indicates a festival is similar to the grand festival circuit mean and, therefore, to the mainstream and many other festivals in a given metadata dimension. A high figure indicates an event that does something very different from the mainstream—and in that sense, contributes to the diversity of the festival circuit. Examples of the latter include specialized festival series such as the Series Mania, which showcases television instead of film content. Importantly, all these diversity metrics are theory-agnostic and convey no value judgment. However, a given low or high diversity metric may be interpreted as positive, negative, valuable, etc., through the lens of a given theory.

We use the same internal diversity approach to calculate the yearly “external diversity” of the whole circuit using all films in all festivals of the year (cf. [60, 61]). This is calculated analogously to internal diversity as the weighted average distance of all films (in a year) from a mean vector. The yearly grand mean vector is weighted the same way as in contributing diversity above (so festivals are treated equally regardless of entry size in the database). We are now equipped for investigating both individual festivals and the festival circuit as a whole.

In the geographic space, both distances and diversity metrics are easily interpretable because the unit is kilometers on the Earth’s surface. For thematic type and language, the latent dimensions are not directly interpretable. However, they can be adjusted to make sense by dividing distances by the absolute maximum possible distance in the underlying latent space (multiplied by 2 for internal and circuit diversity). This scaled distance ranges between 0 and 1. When used in diversity measures, 0 means all films in a festival are exactly the same in the given space (e.g. a festival showcasing only dramas). 1 means a festival showcases the most diverse films possible in a space. When used to calculate the distance between two festivals in space or time, 0 means a festival is identical to another, and 1 means it is as different as possible in the given space.

Naturally, the two complementary measures of internal and contributing diversity autocorrelate to an extent, but this does not pose an issue for our analyses. In the graphs where the two are juxtaposed, this causes a Pareto-like front to appear—this is because it is impossible for a festival to simultaneously have low contributing diversity and high internal diversity. The former would require a festival program to consist of or be near the globally top most frequent category, while the latter would require having two or more very distant categories, which is impossible simultaneously. At the same time, a low internal diversity festival may range freely along the contributing diversity axis.

We use various diversity operationalizations to address the following broad research questions: How does the programming diversity of different film festivals develop over time in terms of film creators’ gender, content themes, film origin geography, and film language? Are there differences between the A- and B-list events?

## Results

This section presents our results in response to the formulated research questions and connects them to findings from previous, primarily qualitative, literature about film festivals. We first reflect on the international film festival circuit as a network of interconnected events. Three festival latent spaces constructed based on film metadata characteristics—content types, origin countries, and languages—are then introduced. Findings about the multifaceted phenomenon of film festival programming diversity based on the three latent spaces and the creator’s gender conclude the section.

### Festival networks

**Fig 2:**
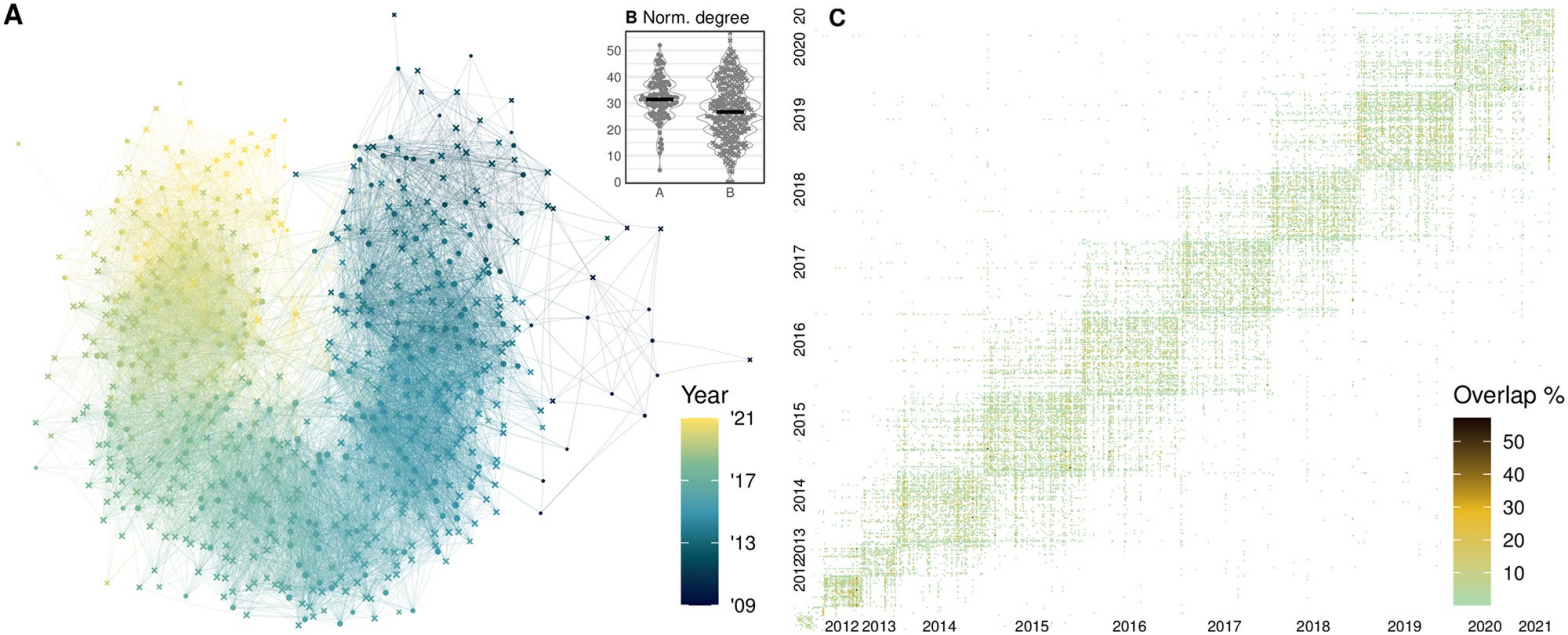
Film festivals analyzed as a network of events connected via overlap in programming. (A) Unipartite spring-embedded event network diagram, different node symbols for A- and B-list festivals, nodes colored chronologically by event year from dark blue to yellow, edges colored by the year of the later event. The longitudinal shape emerges from the dominance of short-range links across time, while a minority of long-range links result in the U shape. (B) Normalized node degree distribution for A- and B-list festivals. (C) The network as an ordered matrix. Color corresponds to program overlap: each row is a festival, colored by the percentage that its program consists of films from other festivals (columns). Clusters along the diagonal correspond to years. Festivals predominantly overlap via films shown in consecutive years, and retrospective festivals appear as network hubs.

We operationalize the expansive Cinando data set as a network of festivals connected via shared films in their programming to illustrate the structure of the international film festival circuit and provide a complementary measure of festival programming diversity. From the bipartite film–festival network featuring 31,989 films programmed at 616 festivals via 41,483 film–festival pairs, we project a unipartite festival network connected via 12,544 festival–festival edges (Fig 2A). The unipartite network is highly interconnected with only four events featuring unique programming without a single entry shared with other festivals. These are different yearly editions of the Series Mania festival, which showcases only television content. The cohesive festival network produced based on the Cinando data differs from the stratified and disunified festival circuit assumed in previous qualitative literature [52, 98].

A clear temporal rhythm in the system emerges when the festivals and their connections are graphed as an ordered matrix (Fig 2C). Most programming is shared between events happening within the same year and, to a lesser extent, between adjacent years. This is explainable by festivals generally aspiring to showcase new content and hard limits by some festivals to only screen films made the same or the previous year [99–101]. Some connections, however, extend across longer periods due to a number of reasons. Retrospective sections of different events might feature the same older productions (e.g. Tokyo Monogatari (1953) in the Classics sections of Berlinale 2013 and Cannes 2018). Films that traveled the circuit upon their release might receive special mentions in later festivals (e.g. Wadjda (2012) included in the Special Program of Busan 2021 after its initial festival run in 2012). Or films might enter the circuit first in the development stage and later as finished productions (e.g. 200 Meters (2020) featured in the talent development section of Berlinale 2015 before its festival run in 2020). These connections between distant years create the curvature in the stress-directed network diagram (Fig 2A), where yellow edges connect to recent festivals and purple ones to older ones. As noted in the Data processing section and visible on the graphs, the festival database has fewer entries in earlier years. We assume this is mainly due to the growth in the usage of the Cinando platform, but could also be due to increasingly more festivals being organized around the world.

As discussed in the Festival networks section, we use normalized degree centrality as a measure of connectedness and as a first diversity proxy. If a festival shares few or no films with other events (low degree, interpretable as high contributing diversity), then it is adding something new to the ecosystem. In contrast, if a festival’s films are also shown elsewhere, then the event might contribute little to no diversity or novelty to the circuit. Still, in reality, and since we do not take festival dates into account, festivals with many connections might act as a launching point for success in future festivals (e.g. being showcased in a high-standing A-list festival) or such festival might add value via an eclectic program that brings together films and audiences that would not otherwise interact. While the A-list festivals have a slightly higher degree centrality on average (Fig 2B), we found no clear increase in the similarity between festival programming during the period (no pronounced gradient of color in the ordered matrix in Fig 2C).

We were also interested if links between programs of festivals via shared films are predictable to some extent and if the intuitions from the graphs could be quantified. We operationalize the data as pairs of all festivals and construct a logistic regression model where the dependent variable is linkage or presence of an edge in the network (616 festivals; 12,544 linked and 176,876 unlinked pairs), predicted by the absolute temporal difference between the events in years (*β* = −1.64, *p* < 0.0001), geographic distance in kilometers (non-significant at *α* = 0.05), and list of the events (A, B), with the difference in lists as the baseline (both being A-list: *β* = 0.35, *p* < 0.0001, both being B-list: *β* = −0.33, *p* < 0.0001); model Nagelkerke pseudo-*R*^2^ = 0.4 or an estimated 40% of variance described. The intercept *e*^0.33^ = 0.72 is the expected odds for a pair of A- and B-list festivals happening in the same place in the same year. With everything else held constant, for every 1-year increase in temporal distance, festival linkage odds decrease by *e*^−1.64^ = 0.19 times. This reflects the 1–2-year circuit pattern discussed above and previous observations [99–101]. In principle, being both A-list festivals relatively increases the odds of linked programming, while being both B-list festivals decreases the odds (both *p* < 0.0001), but this is partly explainable by there being many more B-list festivals across the world, many of which are never linked.

We also constructed a log-linear Poisson regression model to analyze the contribution of the same variables on the extent of festival programming overlap for festivals that share at least one film (*N* = 12, 544). With the dependent variable being the (log) number of shared films, each year in temporal difference decreases overlap by *e*^−0.25^ = 0.78 times (*p* < 0.0001), consistent with the results above. Each increase of 1000km distance is associated with a small effect of 0.98 times decrease of programming share (*p* < 0.0001), relative to the intercept of *e*^0.997^ = 2.7 mean shared films (same intercept logic as above). Both being A-list festivals multiplies overlap by 1.39 times, while both being B-list decreases it by 0.87 (both *p* < 0.0001). The effects being small, this model describes about 3.2% of variance in overlap in festivals that share at least one film (McFadden’s pseudo-*R*^2^, i.e. log-likelihood comparison to null model).

To probe the same question about the extent of overlap between festival programs while taking into account the variable size of festivals as such and festival entries in the database, we construct a linear regression on pairs of festivals that share at least one film (*N* = 25, 088), but predicting program overlap as a percentage of the program of a festival X that is also covered by another festival Y (the intersection of X and Y, divided by the number of films in X, multiplied by 100). This ranges from 0.1% to 57% (Directors’ Fortnight 2015 lists 28 films, 16 of which are also programmed in Toronto 2015). We use a linear model as its assumptions are roughly met. While the previous two models assumed an undirected network, this corresponds to a directed network approach. However, lacking festival dates and taking into account that festival preparation times indeed overlap, we can still only establish precedence by year. The percentage overlap in festivals that share films is predicted by relative time distance, *β* = 0.1, *p* < 0.0001, i.e. festivals in the past are somewhat more likely to have their programs duplicated by future festivals, but each year increases the average overlap by only 0.1% (as indeed most festivals overlap within the same year). The other variables are the same as above, geographic distance (n.s.) and A/B list. Compared to being of different lists, both being A-list festivals increases program overlap by 0.2%; both being B-list is not significant. In summary, as above, the effects are small, reflected by an adjusted *R*^2^ = 0.0005, i.e. here only a minuscule 0.05% variance described. However, this exercise demonstrates that it is possible to operationalize festivals as a network and use their metadata to quantify drivers of connections and overlaps in programming. While we used basic variables like temporal and geographical distance as an example, more informative variables are likely to increase the predictiveness of such models.

### Festival latent spaces

**Fig 3:**
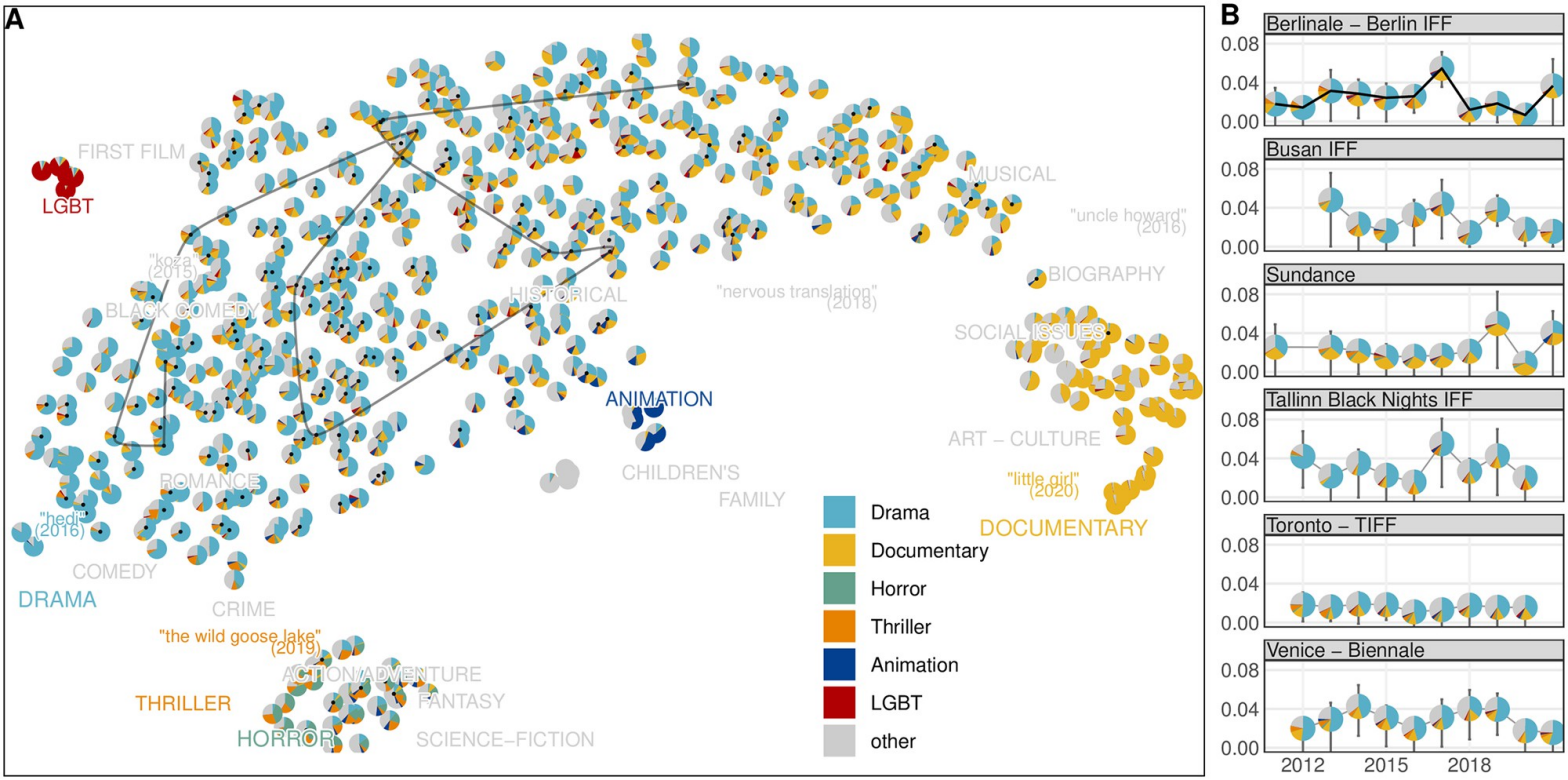
Thematic festival space. (A) Continuous latent space containing thematic content categories (uppercase labels), festivals (circles), and films (a sample shown as lowercase labels) in a shared space, projected using a UMAP dimension reduction. Similar content tags are close to each other, and therefore, also festivals which program films with similar tags. The tiny pie chart circles reflect the distribution of the most frequent discrete categories in a festival program. A-list festivals are highlighted with a black circle. The dark line trajectory marks the latent positions of the Berlinale series, the largest jump being in 2017 toward more documentary but also animation content. (B) Yearly changes in event programming for longer-running festival series. Values near 0 indicate little change, a high value means the event differs from the previous year (bars indicate bootstrapped confidence intervals).

Fig 3A depicts the thematic space of the festival circuit in the form of a UMAP dimension reduction of the vector embeddings of 570 festivals based on 25,016 programmed films (a total of 34,019 film–festival pairs). While any dimension reduction is approximate, and the space appears largely contiguous, the roughly five visible groups do correspond to the industry topology discussed in prior qualitative research. Most festivals primarily program drama content, forming a cluster which compares to general events [2]. The other two clusters fit into the categorization of specialized, thematic genre-based festivals [2]: a group of primarily B-list events (except the A-list Kraków festival) positioned around the documentary, biography, art–culture, and social issues tags correspond to documentary festivals [102]; and a semi-separate cluster in-between action–adventure, fantasy, science fiction, thriller, and horror tags represents what has been referred to as genre festivals [103]. Other clusters align with the identity-based film festival category that targets specific communities or demographics [2], namely queer festivals [104] which surround the LGBT tag, and children’s festivals [105] that fall between family, animation, and children’s tags. Notably, queer and children’s clusters both form solely from different yearly editions of the same festival series (BFI Flare and Annecy Animation, respectively) due to the limited representation of these festival types within our data set. The specialized programming beyond general events maximizes festival agenda-setting effect [15].

In this latent space approach, festivals are means of film vectors, which are, in turn, means of thematic category vectors (see Methods and materials section). It follows that it is possible to quantify the proximity (similarity) between any combination of festivals, films, and categories. As an example, we implement a simple metric of change to quantify how much festival series change in thematic programming over the years by measuring the average distance of the film vectors of the year to the festival mean of the previous year, normalized by the largest possible difference in the thematic latent space (Fig 3B). Change as such can, of course, be conceptualized in a myriad of ways. This variant illustrates how much festivals change their programming year by year, illustrating how some festivals stay fairly consistent in their thematic programming (e.g. Toronto), while others move in the thematic space (e.g. Berlinale, trajectory highlighted in Fig 3A).

**Fig 4:**
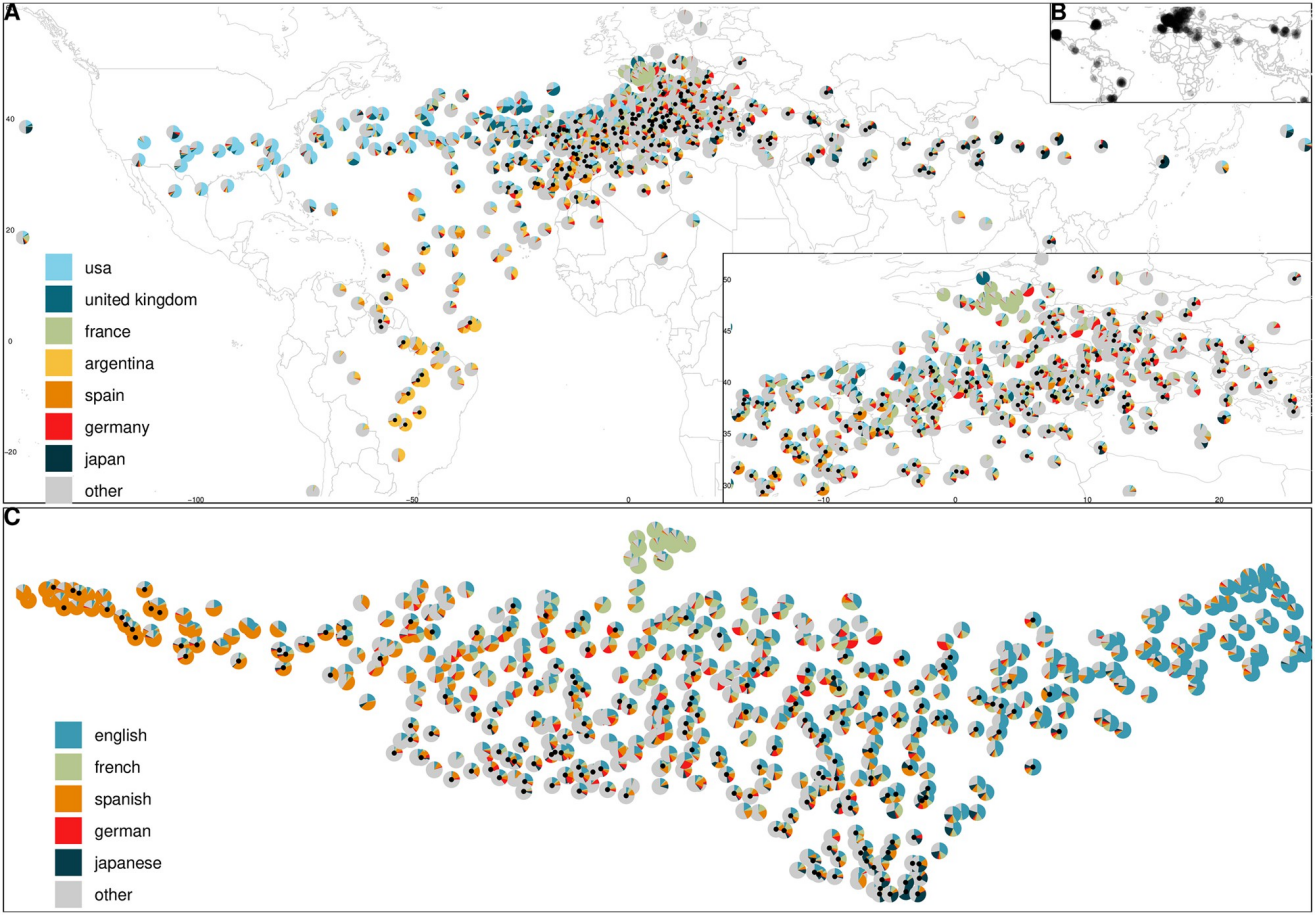
Geographic and linguistic festival spaces. (A) Latent spaces of production geography of festival programs (circles, A-list marked with black dot), with the most frequent countries colored and their shares displayed. The coordinates of this space are the longitude and latitude of the country’s capitals. Each festival is positioned according to the average production country locations in its program (not festival event locations—these are plotted in B), illustrating the European cinema focus of the global festival circuit (further zoomed into in the bottom right corner; note that this neither insets obscure any data points on the map). (B) Festival event locations. (C) Similarly, festivals by the languages represented in their programs; UMAP projection of the latent language similarity embedding. While many festivals are multilingual, there are also groups of Spanish-, English-, and French-focused programs.

Physical global coordinates constitute the geographic space, depicted as a two-dimensional cylindrical topographic projection in Fig 4A. The positioning of festivals in this space marks their geographical focus (not event location marked in Fig 4B), determined as the weighted average of production countries of the programmed films, based on 26,240 films featured at 578 festivals via 51,612 film–festival pairs (all calculations are done on the underlying spherical space). Event clusters with different geographical foci emerging from this visualization are in line with previous qualitative divisions of the international festival circuit into regional sub-circuits. Unsurprisingly given the European origins of our data set and the fact that film festivals started as a European phenomenon [15], events focused on European cinema constitute the largest cluster, although shifted slightly to the South as not many festivals in our data set have a Nordic focus (although there are Haugesund and Göteborg). Another cluster is dedicated to North American cinema and consists of primarily B-list festivals held in English-speaking countries like the UK (e.g. FrightFest), Canada (e.g. Nouveau Cinéma), or the US (e.g. Sundance). A sparse festival cluster focusing on Asian cinema comprises more A- than B-list festivals held mostly within the region (e.g. Tokyo, Busan) and aligns with prior descriptions of this sub-circuit [106, 107]. Another group is festivals programming Latin American cinema situated within the region (e.g. Cine de Lima, Mar del Plata) that are also discussed as a sub-circuit [108]. The geographic space illustrates that while events take place across the world, considerable shares of festival programming are dedicated to European cinema, resulting in a “pull” towards Europe when the production countries of programmed films are put on a map. The strong European focus is a defining feature of the festival circuit and distinguishes it from other forms of film distribution (i.e. theatrical) which is dominated by American productions [15, 52].

The language space positions similar and related languages closer together using an external typological database of linguistic feature vectors. Hence, festivals that program films in the same or similar languages are positioned close together in the language space, depicted as a UMAP dimension reduction in Fig 4C. This space consists of 541 festivals, programming a total of 23,927 films (41,905 film–festival pairs). While the space is fairly contiguous, indicating a lack of fragmentation by language, five clusters with different linguistic specializations may be described. The largest group in the middle consists of multilingual European language films focused festivals, like Cannes and Dok Leipzig. The second-largest cluster of mostly B-list festivals on the right specializes in English-language films (e.g. Sundance, South by South West—SXSW). Another large festival group on the left focuses on Spanish-language cinema (e.g. Ventana Sur, Valdivia). While many festivals program films in French, the one cluster separating from the rest on the top is distinct with predominantly (70–90%) French language programming (e.g. American French Film Festival). The festival group at the bottom programs films in a variety of Asian languages, although Japanese is often the most prominent (e.g. Tokyo, Busan), a tendency explainable by the historical importance of Japanese cinema as the first non-European film culture introduced in Western film festivals [106]. The existence of multiple film festival groups programming primarily non-English language content supports previous observations that film festivals function as resistance spaces to English-language productions which tend to dominate commercial film distribution [109].

### Festival diversity

Using the three latent spaces discussed above, we now compute various festival diversity metrics introduced in the Methods and materials section to evaluate the programming diversity of different festivals over time, compare A- and B-list festivals, and zoom into selected festival series. We also compute gender diversity based on the inferred gender of film creators listed in the database. We evaluate source diversity via creators’ gender and film origin countries and thematic diversity based on film thematic tags and languages. A festival is interpreted as internally diverse in a given metadata dimension when it programs very different films in that dimension. The festival circuit is considered externally diverse when films programmed across all festivals are highly different (we calculate this yearly). Lastly, a festival is considered to contribute to circuit diversity when its programmed films are different from the circuit.

#### Gender diversity

**Fig 5:**
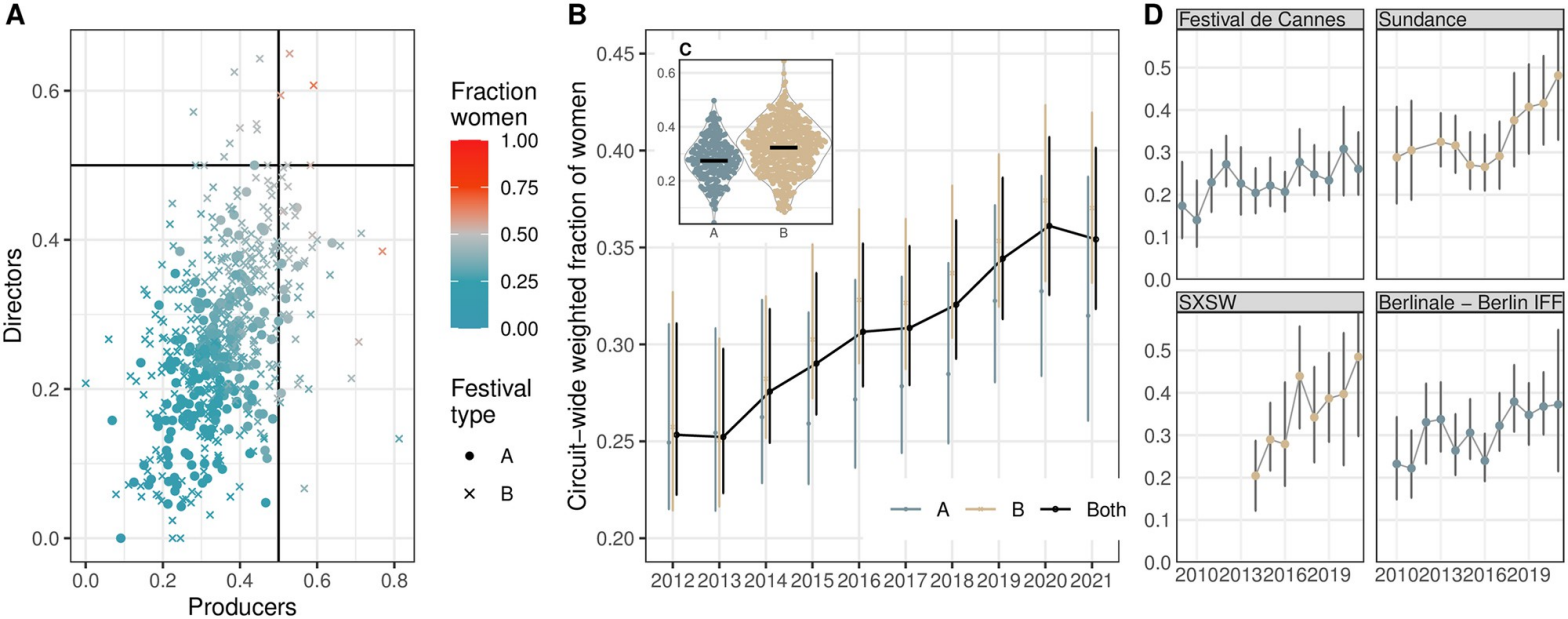
Measuring gender diversity in festival programming. (A) Relationship between creative roles and gender. Each point is a festival, the value reflects the average share of women producers and directors on the films in their programs. Different symbols for A- and B-list festivals, colored by the share of women across both roles. (B) Average share of women directors and producers in the programming of all festivals within a given year (the bars correspond to bootstrapped 95% confidence intervals, taking into account the uncertainty of the gender classifier). (C) Average share of women directors and producers in the programming of all A- and B-list festivals (2009–2021; median for each group is marked with a black bar). (D) Average share of women directors and producers in the programming of four example festival series (Cannes, Sundance, SXSW, and Berlinale).

The internal gender diversity of a festival is calculated as the percentage of women in the key creative roles of the director and producer in its programming, based on 29,610 films featured at 596 festivals via 38,973 film–festival pairs. Fig 5A positions festivals according to the average shares of women producers versus women directors in their programmed films. With the reference lines set at a 50/50% gender representation, the programming of the vast majority of festivals is clearly male-dominated in both creative roles, an inequality noted in previous film festival research [26]. Here and below, we use weighted linear regression to test some individual claims. The weights (number of film entries in a festival entry) are incorporated to take into account the difference between festival entry sizes and the subsequent difference in the uncertainty of aggregated metrics like diversity. In line with observations about the wider film industry on better women’s inclusion among producers [110], across festivals, the percentage of women producers is also higher than women directors (weighted intercept-only linear regression estimating the difference between producer and director fraction: *β* = 0.09, *p* < 0.001, i.e. in the programming of an average festival, there is 9 percentage points more women in producer roles). Some outliers even have a strong majority of women producers (77% in Santiago 2018). B-list festivals are somewhat more gender diverse than A-list events (Fig 5C, weighted regression *β* = 0.06, *p* < 0.001, i.e. 6%-points more women in B-list festivals on average), and festivals programming films with markedly higher shares of women creatives are all on the B-list (e.g. Göteborg 2020, Dok Leipzig 2016). The point estimates of the example B-list event series Sundance and SXSW are close to 50/50% by 2021 (Fig 5D, confidence intervals 33–65 and 30–67%, respectively). This is in line with Sundance’s stance for diversity and previously reported inclusion numbers [111]. Despite the identified gender inequalities, the festival landscape has been changing for the better, as the share of films with women in the two creative roles has increased slightly over the period (Fig 5B, from an estimated share of 24–29% in 2012 to 34–38% in 2021; bootstrapped 95% confidence intervals incorporating the uncertainty of the name-gender classifier).

#### Thematic diversity

**Fig 6:**
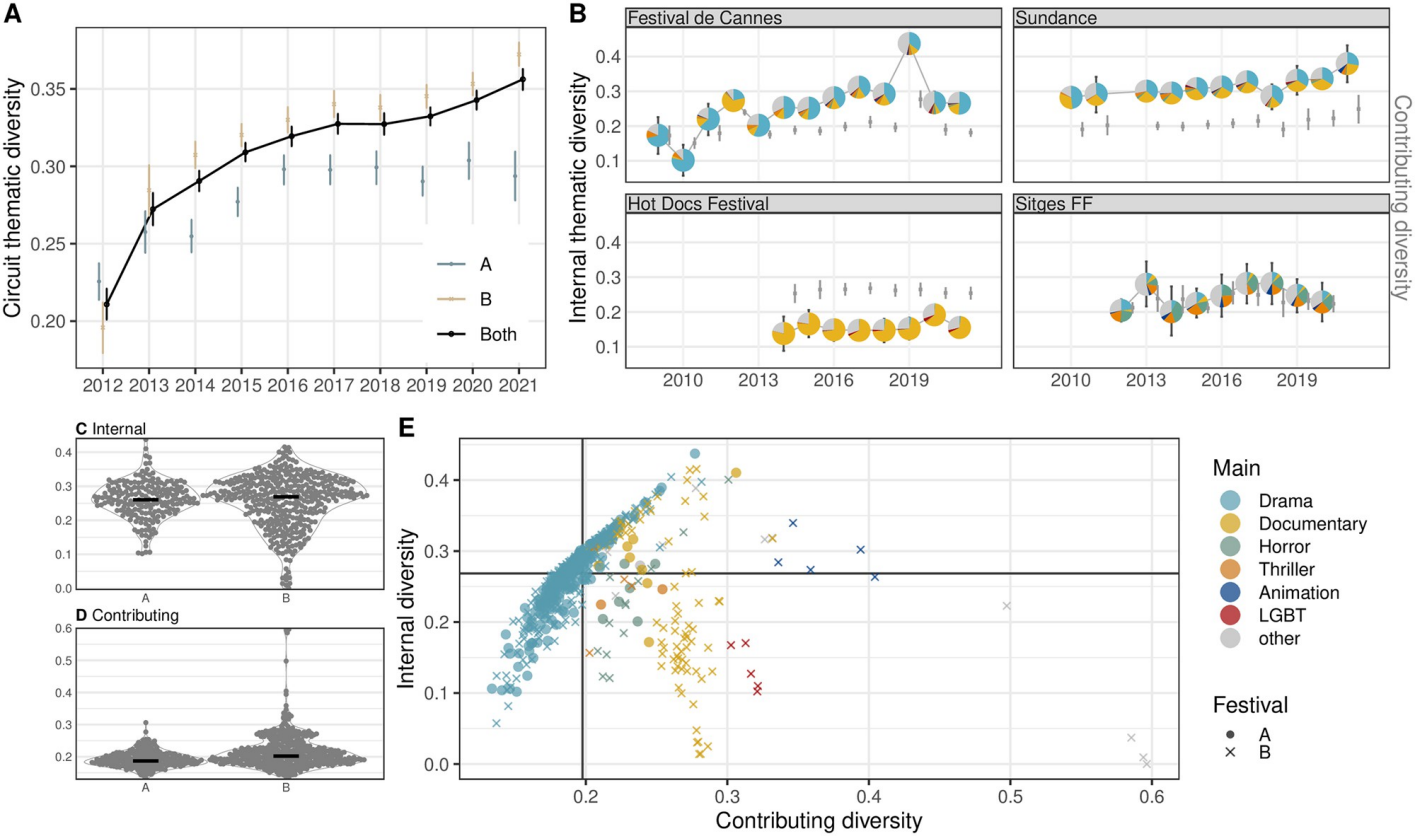
Measuring thematic diversity in film festival programming. (A) Thematic festival circuit diversity; reflects the diversity of the entire circuit year by year. The bars in all plots correspond to bootstrapped 95% confidence intervals. (B) Internal thematic diversity (marked in pie charts) and contributing diversity (marked in gray bars) for four example festival series (Cannes, Sundance, Hot Docs, and Sitges). (C) Internal and (D) contributing diversity of all A- and B-list festivals; the black bars are medians. The former measures how much thematic diversity is expressed in a given festival (a larger number of distinct categories, i.e. larger spread in the latent space, results in higher values). The latter indicates how much the festival diverges from the circuit average, and, as such, is an estimate of how much its existence contributes to overall diversity. Lower values indicate proximity to the mainstream (in this thematic space, drama festivals), higher means the festival is thematically more distinct. (E) Relationship between internal and contributing festival diversity across all events (colored by the most frequent main category).

The film festival circuit has increased in external thematic diversity over the years, although this increase was smaller for the A-list festivals (blue in Fig 6A). Content specialization happens primarily among the B-list events as seen from cases with very low internal diversity (Fig 6C). Yet, some of the B-list events are the major contributors to the overall circuit’s diversity (Fig 6D). When the two metrics are juxtaposed, these are the B-list events specializing in niche content areas, such as documentary (e.g. Sheffield DocFest), queer (e.g. BFI Flare), or television (e.g. Series Mania) (Fig 6E). Most events, however, fall on the continuum where their internal and contributing diversity values are either both low or high. As discussed in the Methods and materials section, this is partially due to the natural autocorrelation of the two metrics; an event cannot have low internal but high contributing diversity. Festivals with both low internal and low contributing diversity offer homogeneous programming similar to the rest of the circuit, adding little to its diversity. These events tend to dedicate most of their programming to drama (e.g. Mumbai). In contrast, festivals with both high internal and high contributing diversity feature heterogeneous programming that adds to the diversity of the circuit. These are genre festivals that screen films of different thematic types, including less common categories of horror and thriller (e.g. Fantastic Fest). From the example series, the highly specialized documentary Hot Docs festival demonstrated lower internal diversity than the genre festival Sitges, open to more fantasy-related content categories (Fig 6B). Sitges, in turn, had lower internal diversity values than the general, mainstream festivals Cannes and Sundance. The contributing diversity of the Hot Docs festival, however, was the highest of the four example series due to its specialization in documentary content less frequent in the circuit (gray bars in Fig 6B).

#### Geographic diversity

**Fig 7:**
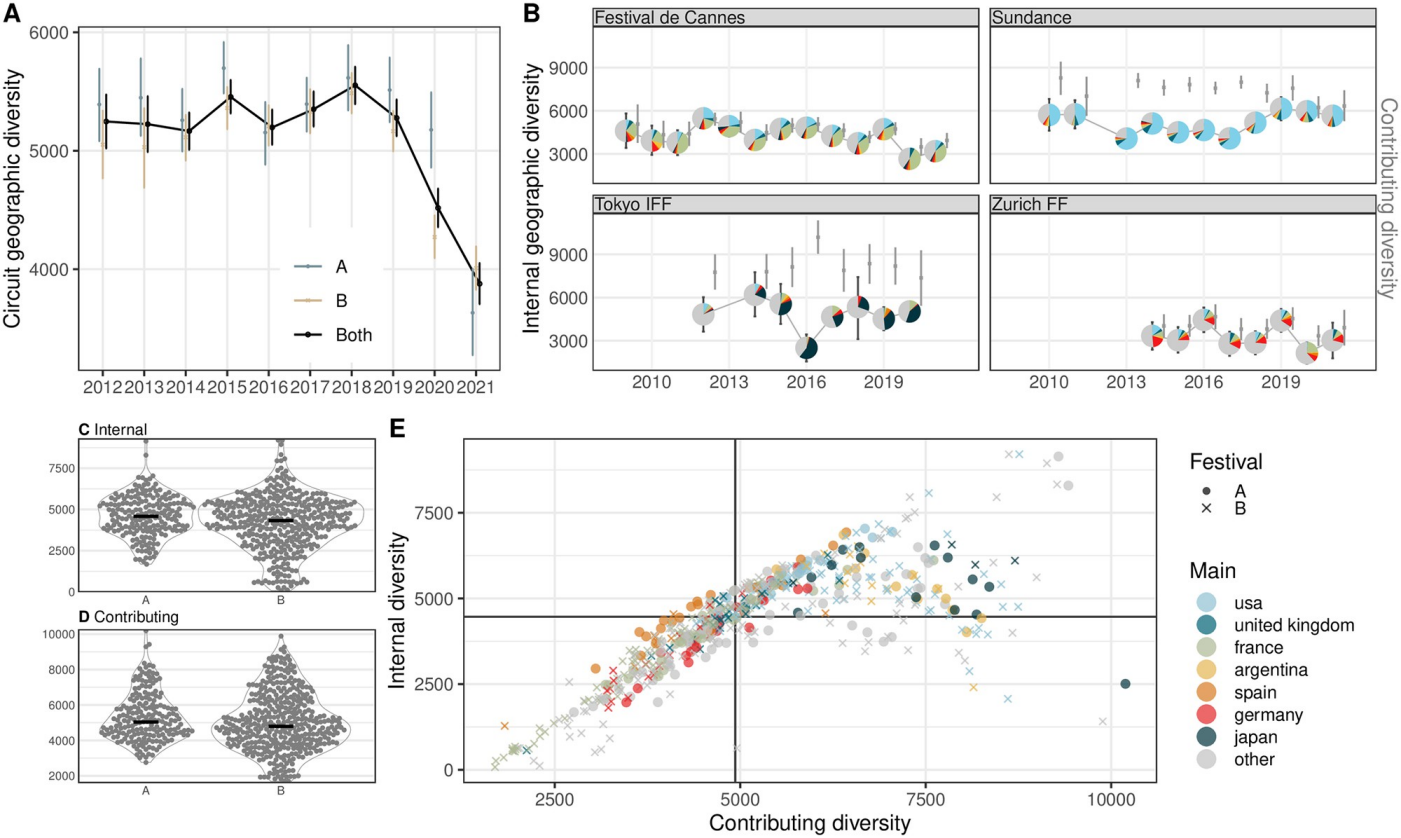
Measuring geographic diversity in film festival programming. (A) Geographic festival circuit diversity; reflects the diversity of the entire circuit year by year. (B) Internal geographic diversity (marked in pie charts) and contributing diversity (marked in gray bars) for four example festival series (Cannes, Sundance, Tokyo, and Zurich). (C) Internal and (D) contributing festival diversity of A- and B-list festivals. The former measures how much geographic diversity is expressed in a given festival: a larger number of distant countries results in higher values. The latter indicates how distant a festival is, in terms of the production countries of its programmed films, from the circuit’s global average latent center (in the geographical space, near South-Western Europe). (E) Relationship between internal and contributing festival diversity across all events.

Aside from other documented effects of the COVID-19 pandemic on film festivals [112, 113], our analysis shows a visible decrease in external geographic diversity during 2020–2021 when the circuit of those festivals which remained in some form operational became more geographically focused (Fig 7A). Given that high-status festivals run geographically unrestricted competitive programs which attract submissions from different world regions, it is surprising to find little difference between the A- and B-lists when it comes to internal (Fig 7C) and contributing (Fig 7D) geographic diversity. Only when the two metrics are contrasted, events with the lowest internal and contributing diversity values are all B-list festivals that specialize in European films which are abundant in the rest of the circuit (e.g. Seville, American French Film Festival) (Fig 7E). In contrast, events with high internal and contributing diversity feature international programming that includes films from less common production countries like Australia (e.g. Melbourne, Sydney festivals). The fewer festivals with low internal but high contributing diversity enrich the circuit by focusing on niche geographical areas, such as films from Latin America (e.g. Santiago) or North America (e.g. Deauville). From the example series, Sundance and Tokyo with a stronger focus on North American and Asian cinema, respectively, contributed more to the geographic circuit diversity than the European-centered Cannes and Zurich, which also became less internally diverse during the COVID-19 pandemic (Fig 7B).

#### Linguistic diversity

**Fig 8:**
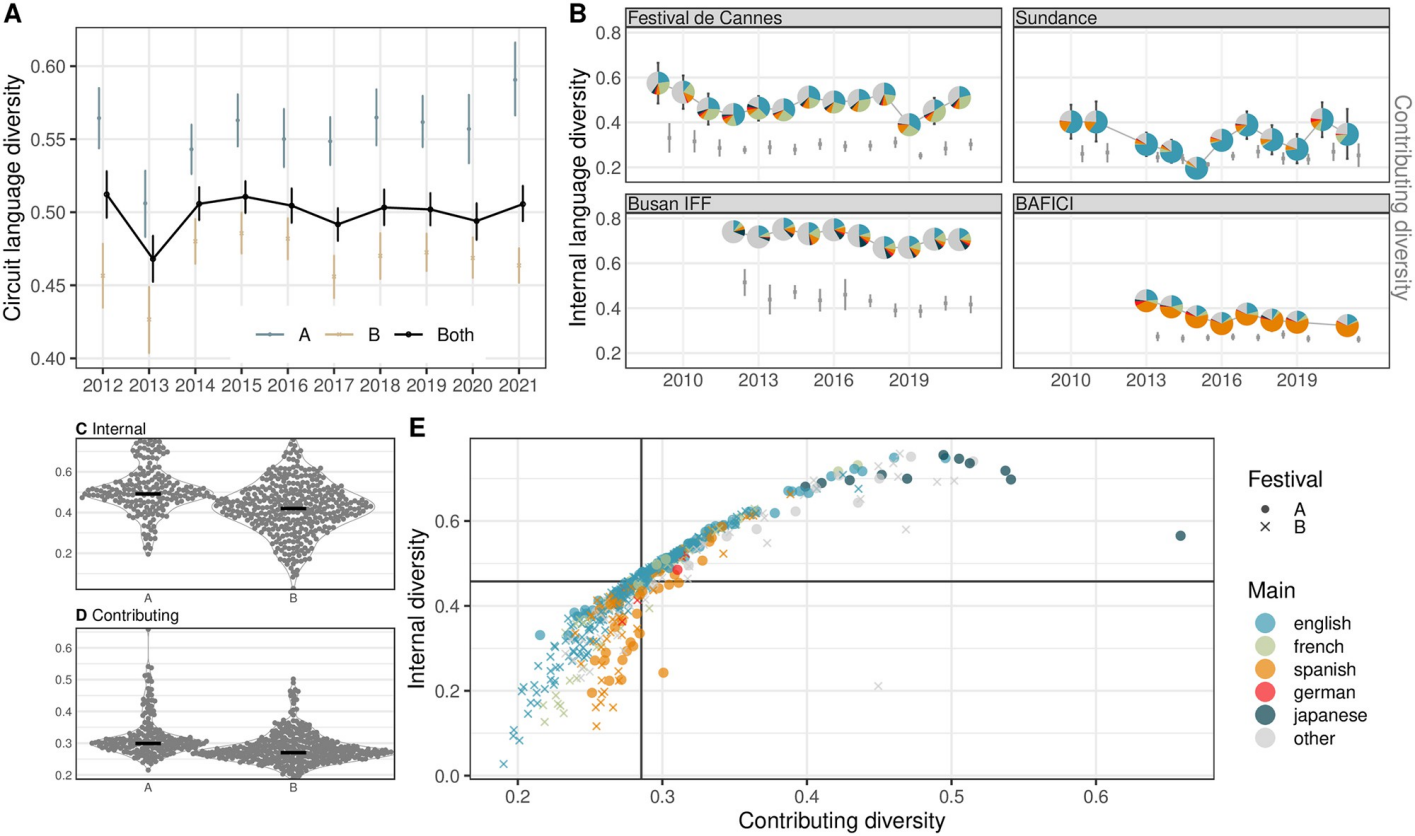
Measuring linguistic diversity in film festival programming. (A) Linguistic festival circuit diversity; reflects the diversity of the entire circuit year by year. (B) Internal linguistic diversity (marked in pie charts) and contributing diversity (marked in gray bars) for four example festival series (Cannes, Sundance, Busan, and BAFICI). (C) Internal and (D) contributing festival diversity for A- and B-list festivals. The former measures how much linguistic diversity is expressed in a given festival. The latter indicates how much the festival differs from the circuit latent average (in the linguistic space: the Indo-European language cluster). Lower values indicate proximity to the mainstream, higher means the festival programs films in less common languages. (E) Relationship between internal and contributing festival diversity across all events.

While the external linguistic diversity of the festival circuit has been roughly stable over the years, the A-list festival group was consistently more linguistically diverse than the B-list (blue in Fig 8A). This might relate to their geographically unrestricted competitive programs which attract international submissions (although this is not visible in geographic diversity); and typically higher budgets that allow hosting such films, including subtitling them for local audiences [2]. Interestingly, across festivals, there is only a mild correlation between linguistic and geographic diversity (Pearson’s *r* = 0.25, *p* < 0.0001, i.e. one describes about 6% variance in the other). The A-list festivals individually are also more internally diverse than B-list events, some of which are highly linguistically specialized (Fig 8C). This is supported by a weighted regression predicting internal diversity by list, with A-list as the baseline (*β* = −0.08, *p* < 0.001, *R*^2^ = 0.13). By showcasing films in various, often less frequently used languages, some of the A-list events are also the main contributors to the linguistic diversity of the circuit (Fig 8D). When the two metrics are juxtaposed (Fig 8E), events with very high internal and contributing diversity values are multilingual A-list festivals dedicated to Asian cinema, showcasing an array of languages different from the European-focused circuit, like Japanese (e.g. Tokyo) or Korean (e.g. Busan). In contrast, festivals with the lowest internal and contributing diversity are B-list events that primarily program films voiced in languages common within the circuit, such as English (e.g. SXSW), French (e.g. American French Film Festival), or Spanish (e.g. Ventana Sur). Events with low internal but high contributing diversity are less common than in the other metadata categories, as few festivals specialize in niche languages. The only one is Fajr 2021 with films in Persian and Kurdish. From the example series, the Asian-focused Busan and the European Cannes had higher internal linguistic diversity than Sundance which programmed primarily English-language films or Buenos Aires—BAFICI which favored content in Romance languages, yet Busan contributed more to the linguistic diversity of the circuit less frequent in Asian languages (Fig 8B).

## Discussion

This section summarizes our key results and grounds them in the theory of public value. We highlight our contribution to both the members of the film industry and researchers, identify the limitations of our study, and outline areas for future research before the next section concludes the paper.

### Summary of key results

We demonstrate how operationalizing festival programming as networks and film and festival metadata as quantitatively comparable metrics can both confirm previous observations and provide novel insights on the film festival circuit and the resulting trends in the film industry. We also provide a solution for properly dealing with metadata where levels in categorical variables may not be equidistant.

With regard to revealing the structure of the international film festival network, we find it to be highly interconnected in contrast to previous claims about a disunified circuit [52, 98]. In terms of dynamics, we find the festival network operates on a clear temporal rhythm where programming overlaps primarily between events held one to two years apart, confirming previous assumptions [99–101]. We also quantify the connections using regression models to predict connectivity and overlap, confirming the temporal pattern observation and demonstrating an application of statistics to predict festival circuit dynamics.

When festivals are fitted into latent spaces according to the metadata of their programmed films, several groups can be roughly observed which to an extent align with industry topologies described in qualitative research. We identify five thematic festival clusters: general [2], documentary [102], genre [103], queer [104], and children’s [105]. The agenda-setting effect of the circuit is likely maximized via programming specialization beyond general events [15]. Four broad geographic festival sub-circuits also emerge: Europe [15], North America, Asia [106, 107], and Latin America [108]. The existence of multiple geographic foci beyond North America and the strong prominence of European content distinguishes the festival circuit from other forms of distribution that are heavily dominated by American productions [15, 52]. We also loosely observe five language-based event groups: English, Spanish, French, a mix of European languages, and a mix of Asian languages. The presence of festival groups programming primarily non-English language content aligns with previous claims that the circuit is more accepting of non-English language content than other forms of distribution [109].

We were particularly interested in understanding how different types of diversity unfold in the film festival circuit. In general, we find no evidence of increased homogenization in festival programming over 2015–2019. We also observe the majority of festivals to be far from gender-equal in terms of directors and producers of the programmed films in line with previous literature [26]. However, the inclusion of films by women in festival programs has increased between 2012–2021. In terms of the external diversity of the festival circuit, we find festival diversities associated with different film characteristics to function in different ways since the circuit has become more thematically diverse, while linguistic as well as geographic origin diversities have not changed much during most of the period, and the latter actually decreased sharply during the pandemic years of 2020–2021.

We were also interested in comparing A- and B-list festivals in terms of societal public value generation. Based on internal festival diversity, we find that the programming of B-list events is often more gender-diverse yet more thematically and linguistically specialized than that of the A-list. Contributing diversity metrics show that via the specialization in niche content areas (i.e. television, children’s, queer, documentary content), B-list events appear to be the main contributors to the thematic diversity of the circuit. However, A-list festivals which tend to have higher internal linguistic diversity are found to also contribute more to the overall circuit’s linguistic diversity, while many B-list events specialize in languages prominent in the circuit. We are surprised to find little difference in geographic diversity between the festival groups, despite many of the high-status A-list festivals running in principle geographically unrestricted competitive programs.

### Relevance to the public value theory

In this paper, we estimated an aspect of public value created by the international film festival circuit, namely value for society. However, serving local audiences with diverse festival programming means also serving the international industries, which explicates how creating value for the industry is also in the public interest [43, 49]. Enabling a diverse and strong institutional system of film production is necessary for the continued production of a diverse cultural sphere. In this regard, festivals are not only important as marketers of films but also as creators of spaces for dialogues, encounters, and networking for international film industries and individuals therein. Not all festivals are equal in this regard, however, since the potential to generate industry value depends on a festival’s positioning within the sector [16]. It has been well established that A-list festivals tend to be more industry-facing events. They are important not only for their competitive programs and related higher status and publicity but also for running film markets in parallel with the main festival as spaces for selling and buying film rights, pitching film projects for funding, and organizing workshops. They constitute nodes in the film industry’s networking practices, which is the value festivals provide for the wider industry. Our study adds to this knowledge by revealing how the programming practices of A-list festivals interlink different linguistic spaces, highlighting the cultural diversities of international filmmaking. In addition, we emphasized the importance of B-list festivals in being more open to films by women and their aggregate effects on increasing the thematic diversity of the festival circuit, enabling films to find their interested audiences internationally. This evidence, in terms of the value that different kinds of festivals provide to the wider international film industries, could be used by national and regional policymakers in designing the festival circuit and finetuning their public remits.

### Contribution to industry professionals and policymakers

That is, the potential use of the methodologies showcased in this paper goes beyond the research context and is foreseeable by both industry professionals and policymakers. The introduced techniques could be adopted by festival programmers interested in tracking the diversity of their programming across different dimensions, including gender, content themes, geography, and language discussed in this paper, but also others. Such analyses would provide an indication of a program’s breadth and disparity between the included productions. It would also help identify any holes in the program’s coverage, such as, for example, a lack of content from certain world regions. This knowledge could, in turn, lead to reconsidering existing film selection practices or, perhaps, inspire the creation of a festival section dedicated specifically to the programming of currently underrepresented films.

Tracking the geographic or linguistic programming diversity could be of particular relevance to (or in the future perhaps even required from) festivals that receive financial support from organizations which aim to increase the cultural diversity of content distributed and consumed across certain regions, such as Europe in the case of the European Commission. For example, for over 30 years, the Creative Europe MEDIA program has been granting financial support to foster the circulation of European works via the network of festivals that “screen a significant proportion of non-national European films” [114]. Methodologies proposed in this paper could also be potentially used by policymakers for evaluating the historical programming records, to check the eligibility of festivals applying for such funding and yearly programming of the beneficiary festivals to track their progress.

### Limitations of our study

While the Cinando database used as the main data source for this paper offers the most extensive, global, longitudinal information available on the programming of various international film festivals, it has certain limitations, and our results should be interpreted in light of those limitations. The film festival system adheres to its power structures where the Cannes film festival, a European organization that established the Cinando database, occupies the top tier [4]. Given Cannes’ geographical positioning and selective programming procedures, the Cinando database might have historically underrepresented emerging and non-European filmmakers or events that are smaller and located outside Europe. However, the Cinando platform has grown to feature and service an array of festivals from across the hierarchy and the world over the years, which has likely also led to the diversification of its database. In fact, festivals accredited by FIAPF which we consider A-list events are in the minority of our sample. While we presented here the entirety of the database to the extent that we managed to operationalize it, we refrain from making inferences about the first few years therein due to very few initial festivals. Another limitation is that the Cinando data on event programming only covers films accepted to festivals. Lacking information on unsuccessful entries might, therefore, create a certain survivorship bias (cf. [115]) in subsequent inferences and summaries. Since our data essentially describe only successful films, we refrain from conclusions such as success factors for films in the festival circuit. The outlined limitations considered, the Cinando database remains a unique resource that for the first time enables quantitative, longitudinal, and at-scale analysis of the international film festival network.

### Future research

We hope that the demonstration of both the data and methods of network analysis and latent embeddings in this paper encourages and inspires future research using quantitative techniques into film festival data sets, supported by the theoretical frameworks of public value and diversity. In particular, we propose quantifying further aspects of public value creation within the film festival circuit beyond the value generated for society via diverse festival programming. These aspects could include value for individuals and industry [49], operationalized via film professional networks or sales markets. We also hope that our quantifications and visualizations of the large-scale, longitudinal international film festival data available as an interactive online dashboard (see S4 Fig) will encourage further exploration and case studies on aspects of the data. This approach could also be developed into a functional film festival diversity indicator for the circuit as a whole and each individual festival. Similar to Altmetric for scholarly publications or the compound measure of the Human Development Index, this could be updated in regular intervals and have a broad impact on the film festival industry.

Importantly and more generally, the methods we used here are applicable to a wide range of metadata types, both those of festivals and of the programmed content. We used production countries and languages, but other cultural variables, if available, could be included to measure cultural diversity or complexity more directly (as languages and countries do not always align with cultural borders). In some cases, externally sourced latent spaces can be used to embed non-equidistant categorical variables (the geographic coordinates for production countries being the simplest example). Where this is not possible, such as in the thematic type example above, spaces can be inferred by leveraging co-occurrence information analogously to the distributional semantics application of word embeddings. This includes film content: frames or stills from films could be embedded using visual machine learning methods, or their synopses (or scripts, subtitles, etc.) using topic or text embeddings. The latter is a more exciting opportunity given recent rapid advances in applying generative large language models such as GPT-4 as on-demand classifiers to textual data [116, 117]. Festivals can then be compared, in a shared latent space, by the visual or topical similarity of their programmed films. We treated each metadata dimension separately, but spaces can also be concatenated (and weighted, if necessary) to produce joint embeddings [118, 119].

Future work could also advance the proposed network analysis methods, e.g. by using directed event network graphs to trace the temporal flow of films through the circuit (if event dates are available) or connect festivals not by programming but by the involved filmmakers, market representatives, shared genres, etc. While our analyses have been festival-centric, future research could use the same or similar methods to focus the analysis on films, connecting them via participation across festivals or positioning them across analogous metadata spaces (see example in S3 Fig).

Besides being applicable to other film festival-related data, the methods developed and showcased here are expected to be general enough to be applicable to the quantification and study of any planned or non-planned events, cultural or otherwise. The core of the contribution boils down to this:

1. Events are operationalized as quantitatively comparable units (e.g. distributions or vectors), using their metadata and/or the data characterizing their programs or participation (programs of films, film market participants, lineups of bands, rosters of artists or authors, visitor demographics, etc.). The variables can be (a mix of) metadata like the gender of film producers, musical genre of a band; or operationalization of the content itself (as discussed above, e.g. visual embeddings).
2. If some variables are categorical, and non-equidistance is a problem, then they are embedded in continuous (latent) spaces that ensure the distance between categories is taken into account in comparison.
3. The latent spaces may be externally sourced or inferred directly from the data.
4. The continuous representation allows for direct comparison of events, measurement of change dynamics, qualitative interpretation, operationalization of diversity metrics, and by proxy, of generated public value.

This means that any socio-cultural event series or circuit can be analyzed this way if such data are available or can be inferred. This includes any festivals, exhibitions, fairs, political summits, sporting tournaments, etc. The focus of this general cultural event analytics approach, as described above, is the operationalization and comparison of events primarily through their content or substance. This is slightly different from the more general statistical “event analytics” and “event identification” (of data streams of any events or processes in societies and organizations; see [120–122]), the related, economics-focused “event studies” [123], event studies in the context of management and tourism research [5, 35, 38], the broader “cultural analytics” [124, 125], anthropological “event analysis” [126], “critical event studies” (more concerned with theoretical criticism, participant experiences, and social impact [127]), and the broader, interdisciplinary, but often behavior and economics focused, leisure and tourism studies [128, 129]. Taking one step further, and working towards embedding different event types in comparable shared latent spaces, could pave the way towards a more encompassing socio-cultural “event science”, which in turn could inform the future discourse on diversity and public value generation, policymaking, and event planning.

## Conclusions

This paper presented a quantitative analysis of the international film festival circuit that has never been done before due to limited access to film festival data for academic research and typically small-scale, qualitative foci in film festival studies. Our application of network analysis and vector embedding techniques based on film metadata offers a novel method for the computational exploration of film festival data sets. Such an approach enables asking specific quantitative questions and testing hypotheses, quantifying trends, and making market predictions for the future. Upon quantifying the global festival circuit, we find our results support many previous observations while also providing novel insight into the dynamics of this highly complex international ecosystem. Finally, we suggest that the data-driven methodology adapted and developed here is also applicable for the systematic analysis of any social or cultural events where sufficient data is available.

## Supporting information

S1 Table Results of the festival title disambiguation process. (PDF)

S2 Table Festival name glossary. The table only lists festivals referenced in the text using shortened titles and not all festivals in the sample.
(PDF)

S1 Fig Linguistic latent space of languages in the Cinando database. Language names are standardized for matching with the linguistic vectors database discussed in Methods; UMAP projection. Proximity indicates similarity: e.g. Indo–European languages are all close together. This multidimensional space is, however, difficult to project well into 2D, as some languages are linguistically unrelated and thus far from all other languages.
(PDF)

S2 Fig Latent space of thematic categories in the Cinando database. UMAP dimension reduction of the full vector space. Proximity indicates similarity, as inferred from co-occurrence data in the Cinando database.
(PDF)

S3 Fig Thematic latent space of all films from our subset of Cinando festivals. Films are embedded first in a latent thematic space and projected to 2D here using UMAP. Many films have just one thematic tag, like drama or documentary (the two large clusters), while others have multiple tags, and their position corresponds to the average of the corresponding tag vectors.
(PDF)

S4 Fig Three screenshots from the interactive supplementary dashboard. The graphs in this paper are also available as an interactive dashboard at https://andreskarjus.github.io/cinandofestivals. The graphs can be zoomed in, and hovering over data points reveals further details.
(PDF)
