# Supplementary material for: Quantifying the global film festival circuit: Networks, diversity, and public value creation
Source: PLoS One. 2024 Mar 6;19(3):e0297404. doi: 10.1371/journal.pone.0297404 (PMC10917328; doi:10.1371/journal.pone.0297404)
Supplement: S1 Table — (PDF) [file pone.0297404.s001.pdf]

	Total film–festival pairs	Film–festival pairs with a main festival		Film–festival pairs without a main festival	
	N	*N*	%	*N*	%
**Original data**	183,865	84,971	46%	98,894	54%
**After 1st cleaning iteration**	183,865	119,404	65% (+19%)	64,461	35%
**After 2nd cleaning iteration**	183,865	126,956	69% (+4%)	56,909	31%
