# Supplementary material for: Quantifying the global film festival circuit: Networks, diversity, and public value creation
Source: PLoS One. 2024 Mar 6;19(3):e0297404. doi: 10.1371/journal.pone.0297404 (PMC10917328; doi:10.1371/journal.pone.0297404)
Supplement: S2 Table — The table only lists festivals referenced in the text using shortened titles and not all festivals in the sample. (PDF) [file pone.0297404.s002.pdf]

Short title	Full title
Annecy Animation	Annecy International Animation Film Festival
Cannes	Festival de Cannes
Cine de Lima	Festival de Cine de Lima
BAFICI	Buenos Aires International Festival of Independent Cinema
Berlinale	Berlin International Film Festival (Berlinale)
BFI Flare	BFI Flare: London LGBTIQ+ Film Festival
Busan	Busan International Film Festival
Deauville	Deauville American Film Festival
Fajr	Fajr International Film Festival
Göteborg	Göteborg Film Festival
Haugesund	Norwegian International Film Festival (Haugesund)
Hot Docs	Hot Docs Canadian International Documentary Festival
Kraków	Kraków Film Festival
Mar del Plata	Mar del Plata International Film Festival
Melbourne	Melbourne International Film Festival (MIFF)
Mumbai	Jio MAMI Mumbai Film Festival
Nouveau Cinéma	Festival du Nouveau Cinéma de Montréal (FNC)
Santiago	Santiago International Film Festival (SANFIC)
Seville	Seville European Film Festival
Sitges	Sitges International Fantastic Film Festival of Catalonia
Sundance	Sundance Film Festival
SXSW	South by Southwest (SXSW)
Sydney	Sydney Film Festival
Tallinn	Tallinn Black Nights Film Festival (PÖFF)
Tokyo	Tokyo International Film Festival
Toronto	Toronto International Film Festival
Valdivia	Valdivia International Film Festival
Venice	Venice International Film Festival (Biennale)
Zurich	Zurich Film Festival
