# Supplementary figures and images for: Quantifying the global film festival circuit: Networks, diversity, and public value creation
Source: PLoS One. 2024 Mar 6;19(3):e0297404. doi: 10.1371/journal.pone.0297404 (PMC10917328; doi:10.1371/journal.pone.0297404)

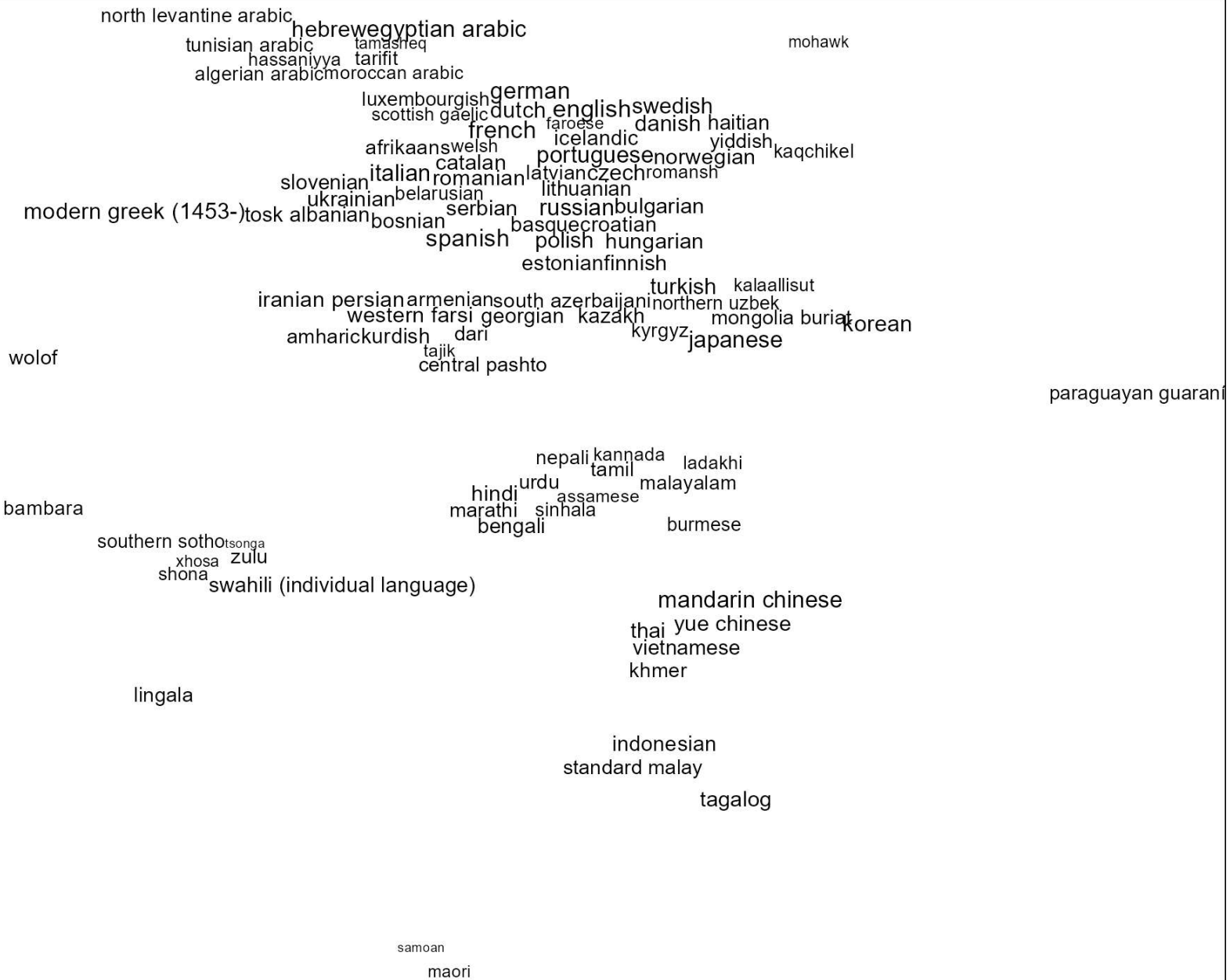

Supplement: S1 Fig — Language names are standardized for matching with the linguistic vectors database discussed in Methods; UMAP projection. Proximity indicates similarity: e.g. Indo–European languages are all close together. This multidimensional space is, however, difficult to project well into 2D, as some languages are linguistically unrelated and thus far from all other languages. (PDF) [file pone.0297404.s003.pdf]

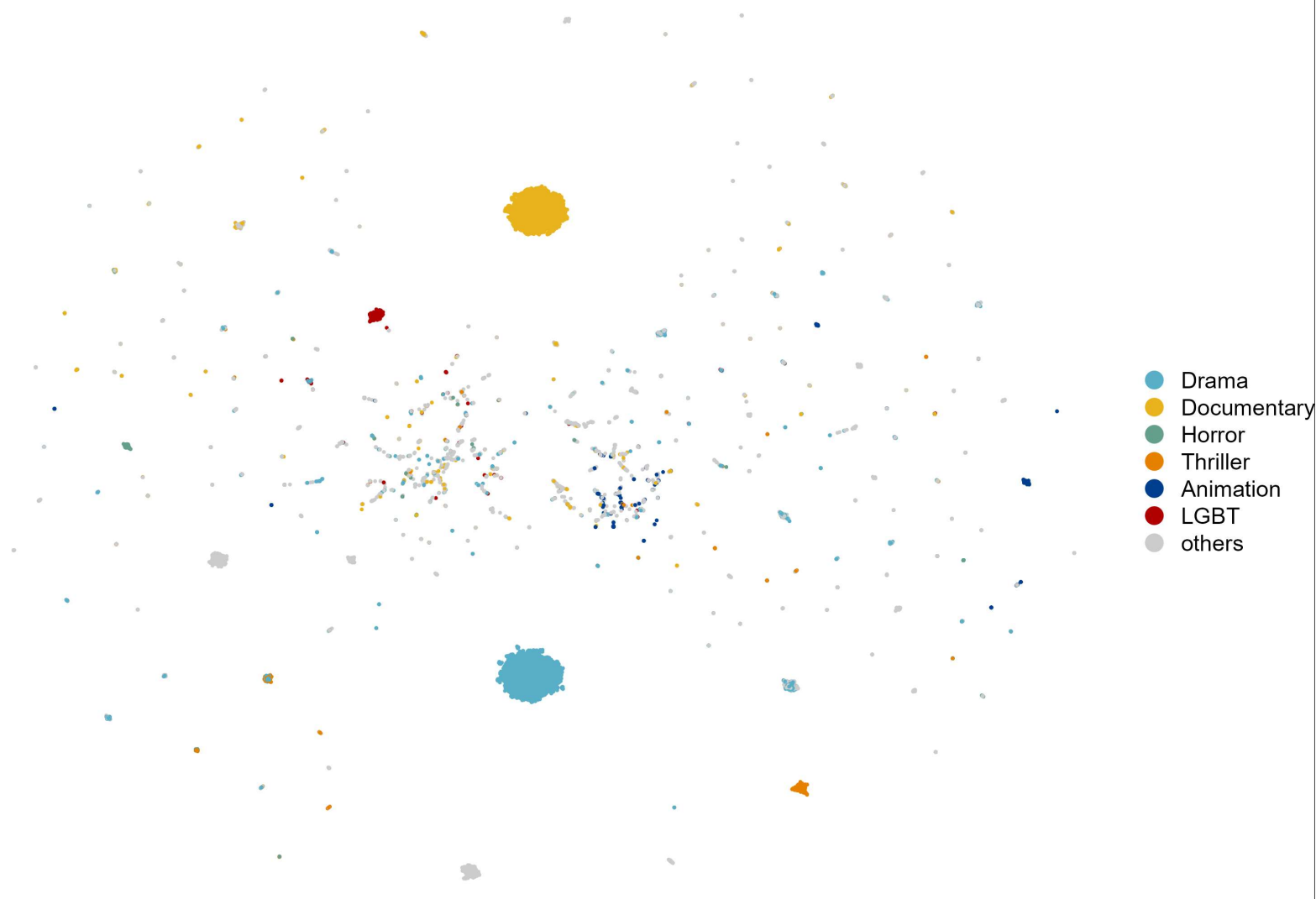

Supplement: S3 Fig — Films are embedded first in a latent thematic space and projected to 2D here using UMAP. Many films have just one thematic tag, like drama or documentary (the two large clusters), while others have multiple tags, and their position corresponds to the average of the corresponding tag vectors. (PDF) [file pone.0297404.s005.pdf]
