# Supplementary material for: Quantifying the global film festival circuit: Networks, diversity, and public value creation
Source: PLoS One. 2024 Mar 6;19(3):e0297404. doi: 10.1371/journal.pone.0297404 (PMC10917328; doi:10.1371/journal.pone.0297404)
Supplement: S2 Fig — UMAP dimension reduction of the full vector space. Proximity indicates similarity, as inferred from co-occurrence data in the Cinando database. (PDF) [file pone.0297404.s004.pdf]

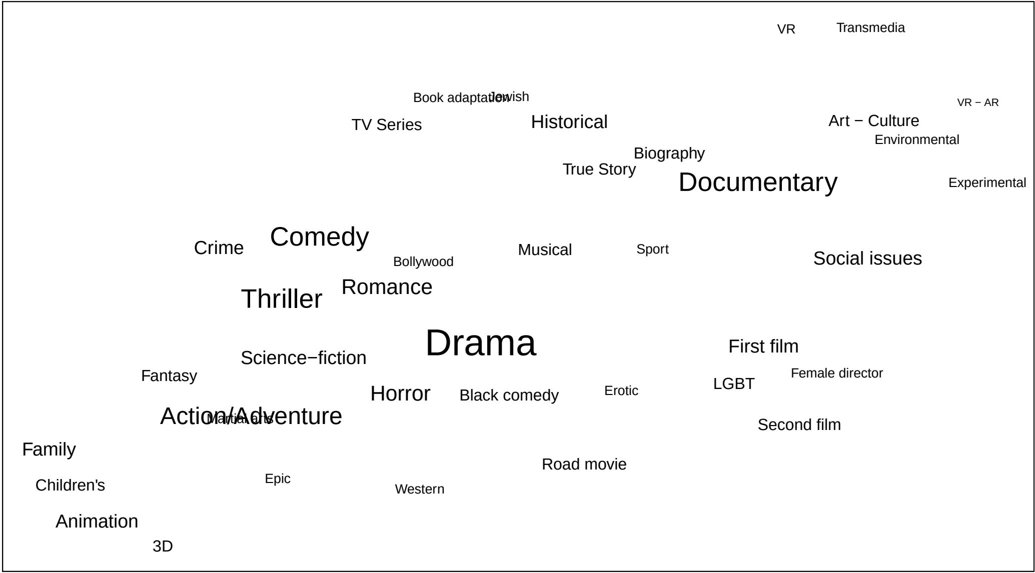
VR
Transmedia
Book adaptation
Jewish
VR − AR
TV Series
Historical
Art − Culture
Environmental
Biography
True Story
Documentary
Experimental
Crime
Comedy
Musical
Sport
Bollywood
Social issues
Thriller
Romance
Drama
First film
Science−fiction
Fantasy
Female director
Horror
Black comedy
Erotic
LGBT
Action/Adventure
Martial arts
Second film
Family
Road movie
Epic
Children's
Western
Animation
3D
