# Supplementary material for: Quantifying the global film festival circuit: Networks, diversity, and public value creation
Source: PLoS One. 2024 Mar 6;19(3):e0297404. doi: 10.1371/journal.pone.0297404 (PMC10917328; doi:10.1371/journal.pone.0297404)
Supplement: S4 Fig — The graphs in this paper are also available as an interactive dashboard at https://andreskarjus.github.io/cinandofestivals. The graphs can be zoomed in, and hovering over data points reveals further details. (PDF) [file pone.0297404.s006.pdf]

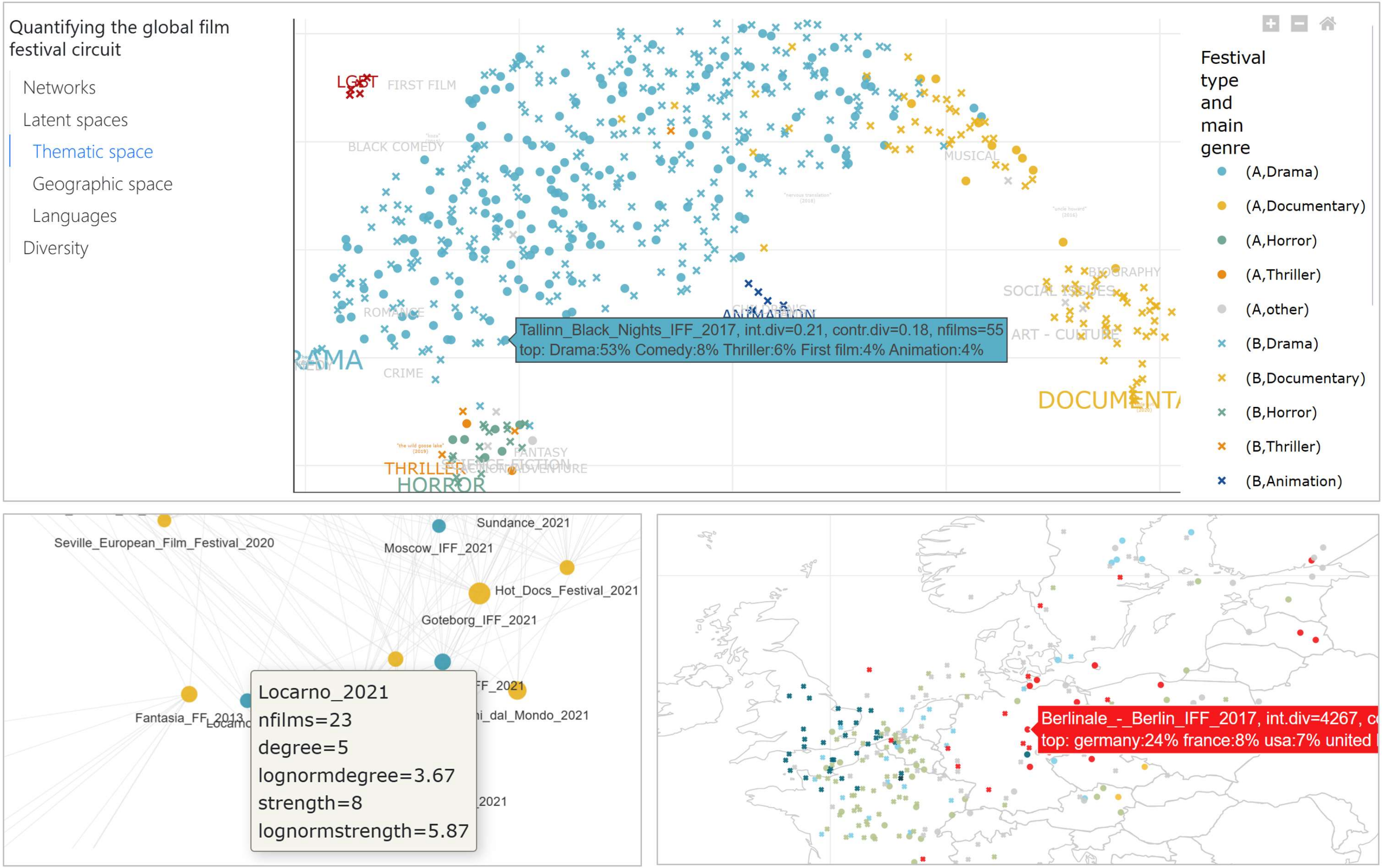
Quantifying the global film festival circuit
Networks
Latent spaces
Thematic space
Geographic space
Languages
Diversity
LGBT
FIRST FILM
BLACK COMEDY
ROMANCE
CRIME
MUSICAL
ANIMATION
BIOGRAPHY
SOCIAL ISSUES
ART - CULTURE
DOCUMENTA
FANTASY
SCIENCE FICTION
THRILLER
HORROR
Tallinn_Black_Nights_IFF_2017, int.div=0.21, contr.div=0.18, nfilms=55
top: Drama:53% Comedy:8% Thriller:6% First film:4% Animation:4%
Festival type and main genre
(A,Drama)
(A,Documentary)
(A,Horror)
(A,Thriller)
(A,other)
(B,Drama)
(B,Documentary)
(B,Horror)
(B,Thriller)
(B,Animation)
Seville_European_Film_Festival_2020
Sundance_2021
Moscow_IFF_2021
Hot_Docs_Festival_2021
Goteborg_IFF_2021
Fantasia_FF_2013
Locarno_2021
nfilms=23
degree=5
lognormdegree=3.67
strength=8
lognormstrength=5.87
Berlinale_-_Berlin_IFF_2017, int.div=4267,
top: germany:24% france:8% usa:7% united
